# LncRNA GAS5 Knockdown Mitigates Hepatic Lipid Accumulation *via* Regulating MiR-26a-5p/PDE4B to Activate cAMP/CREB Pathway

**DOI:** 10.3389/fendo.2022.889858

**Published:** 2022-07-26

**Authors:** Shizan Xu, Yajie Wang, Zhengyang Li, Qian Hua, Miao Jiang, Xiaoming Fan

**Affiliations:** Department of Gastroenterology, Jinshan Hospital of Fudan University, Shanghai, China

**Keywords:** non-alcoholic liver disease, hepatic lipid metabolism, GAS5, miR-26a-5p, phosphodiesterase (PDE) 4B, cAMP/CREB pathway

## Abstract

**Objective:**

Non-alcoholic fatty liver disease (NAFLD) can be attributed to the dysregulation of hepatic lipid metabolism; however, its cellular and molecular mechanisms remain unclear. This study aims to explore the effect of long non-coding RNA growth arrest specific 5 (GAS5) on hepatic lipid metabolism in fatty liver models.

**Methods:**

Obese mice, high fat diet-fed mice and free fatty acid-stimulated cells were used for GAS5 expression detection. GAS5 overexpression or knockdown models were established to elucidate the regulatory function of GAS5 in *de novo* lipogenesis (DNL) and mitochondrial function. Bioinformatic analyses and dual luciferase assays were used to investigate the interaction between GAS5, miR-26a-5p and phosphodiesterase (PDE) 4B. The involvement of the cyclic adenosine monophosphate (cAMP)/cAMP-response element-binding protein (CREB) pathway was evaluated using H89 and forskolin treatment.

**Results:**

GAS5 was activated *in vitro* and *in vivo* fatty liver models. Knockdown of GAS5 reduced lipid droplet accumulation, DNL associated enzymes and preserved mitochondrial function, while GAS5 overexpression exacerbated hepatic lipid accumulation. Mechanistically, GAS5 sponged miR-26a-5p to increase PDE4B expression and subsequently modulated DNL and mitochondrial function *via* the cAMP/CREB pathway.

**Conclusion:**

Downregulation of GAS5 can activate the cAMP/CREB pathway through miR-26a-5p/PDE4B axis to mitigate hepatic lipid accumulation. This study provides evidence that downregulation of GAS5 may be a potential therapeutic option for the treatment of NAFLD.

## Introduction

Along with obesity and metabolic syndromes, non-alcoholic fatty liver disease (NAFLD) is among the most common chronic liver disease worldwide, with a global prevalence of approximately 25% (1, 2). NAFLD comprises a spectrum of fatty liver disorders, ranging from simple steatosis to non-alcoholic steatohepatitis (NASH), which can advance to liver cirrhosis and hepatocellular carcinoma (HCC) (3, 4). Abnormal hepatic lipid metabolism, especially excessive hepatic triglyceride (TG) accumulation, leads to NAFLD development (5, 6). Therefore, elucidating the cellular and molecular mechanism of hepatic lipid metabolism in fatty liver is crucial.

*De novo* lipogenesis (DNL), esterified free fatty acids (FFA) and excessive dietary intake contribute to the hepatic TG accumulation (7), and increased DNL plays a dominant role in NAFLD development (6). The catalysis of DNL-associated enzymes including adenosine triphosphate citrate lyase (ACLY), acetyl-coenzyme A carboxylase (ACC), and fatty acid synthase (FAS), leads to the conversion of citrate into fatty acid, which is eventually esterified to form TGs and other lipids (7). In addition to DNL, mitochondrial dysfunction is also involved in the pathogenesis of NAFLD; therefore, mitochondrial-related molecules could be used as potential targets for NAFLD treatment (8, 9). Peroxisome proliferator-activated receptor γ coactivator-1α (PGC-1α) plays an eventful role in the regulation of mitochondrial biogenesis and oxidative metabolism (10) and is highly expressed in mitochondrial-rich tissues, such as liver, brown fat and skeletal muscle (11). Moreover, owing to an upregulated lipogenic gene expression and a limited mitochondrial respiratory capacity, severe hepatic steatosis was observed in PGC-1α knockout mice (12).

Long non-coding RNA (lncRNA) is commonly defined as a class of transcript that has no protein coding capacity, with a length of over 200 nucleotides (13). Recently, lncRNAs have been increasingly reported to influence the development of various human diseases, including NAFLD. For example, lncRNA Gm15622 was confirmed to activate the hepatic DNL by sponging miR-742-3p and aggregated hepatic lipids accumulation (14). But fewer studies had linked lncRNAs to mitochondrial oxidative function in fatty liver compared to DNL. LncRNA growth arrests specific 5 (GAS5) was firstly isolated from the fibroblasts of a growth-arrested mouse, wherein the expression of GAS5 was significantly upregulated post-transcriptionally (15). Subsequently, GAS5 was identified as the 5’-terminal oligopyrimidine (5’TOP) class of genes that are clustered in messenger ribonucleoprotein (mRNP) particles when cell growth is arrested (16). Regarded as a tumor suppressor, GAS5 regulates cellular proliferation, invasion and metastasis in various types of human tumors (17, 18). Additionally, overexpressed GAS5 can reduce liver fibrosis by inhibiting the activation of stellate cells and reducing the accumulation of hepatic collagen (19). GAS5 has been recently reported to be upregulated in liver tissues and serum of patients with NAFLD (20); however, its function and underlying mechanism remain unclear.

MicroRNA (miRNA) is another group of non-coding RNAs, with 21–25 nucleotides, that work as post-transcriptional suppressors of targeted genes by facilitating mRNA degradation (21, 22). Functions of various miRNAs, including miR-26a, miR-34a and miR-214-3p, have been investigated in NAFLD (21–23). Overexpression of miR-26a has been reported to ameliorate hepatic steatosis by directly inducing eukaryotic initiation factor 2α (EIF2α) in mice fed with high-fat diet (23). Interestingly, the sponging of GAS5 with miR-26a-5p has been observed in degenerative nucleus pulposus cells, diabetic cardiomyopathy and laryngeal squamous cell carcinoma (24–26). Hence, the potential of GAS5 as a competing endogenous RNA (ceRNA) for miR-26a-5p in fatty liver is worth exploring.

PDE4B is a member of the Phosphodiesterase-4 (PDE4) family, whose inhibition regulates the cyclic adenosine monophosphate (cAMP) homeostasis and promotes the hepatic carnitine palmitoyl transferase 1α (CPT-1α) expression in alcohol-induced steatosis (ALD) (27). Given the similar pathophysiological changes between NAFLD and ALD, PDE4B/cAMP could be involved in the development of NAFLD.

This study verifies the expression of GAS5 in *in vivo* and *in vitro* fatty liver models and explores the function and mechanism of GAS5 in hepatic lipid metabolism.

## Methods and Materials

### Materials and Reagents

High fat diet (HFD) with 60% kcal from fat and normal chow diet (ND) with 9.7% kcal from fat were purchased from Biotech HD Co., Ltd. (Beijing, China) and Future Biotech Co., Ltd. (Beijing, China), respectively. Oleic acid (OA), palmitic acid (PA), Dulbecco’s Modified Eagle Medium (DMEM), bovine serum albumin (BSA), high-performance liquid chromatography (HPLC)-grade formic acid, HPLC-grade ammonium formate and ammonium acetate (NH_4_AC) were purchased from Sigma-Aldrich (MO, USA). Foetal bovine serum (FBS) was obtained from Biological Industries (Israel). H89, forskolin, lipid peroxidation malondialdehyde (MDA) assay kit, superoxide dismutase (SOD) assay kit and ATP detection kit were purchased from Beyotime (Shanghai, China), whereas the triglyceride (TG) assay kit was purchased from Nanjing Jiancheng Institute of Biotechnology (Nanjing, China). Lipofectamine 3000, enhanced chemiluminescence (ECL) chromogenic reagent, mass spectrometry (MS)-grade methanol, MS-grade acetonitrile, HPLC-grade 2-propanol and ammonium hydroxide (NH_4_OH) were purchased from Thermo Fisher Scientific (MA, USA). Acetonitrile was purchased from Merck (Darmstadt, Germany). Haematoxylin and eosin (HE), oil red O staining kits and DAPI were obtained from Service Biology (Wuhan, China). Phosphate buffered saline (PBS) and radioimmunoprecipitation assay (RIPA) lysis buffer were purchased from KeyGEN (Nanjing, China). An enzyme-linked immunosorbent assay (ELISA) kit for cAMP analysis was purchased from Chen Gong Biotechnology Co. Ltd. (Shanghai, China). RNA extraction kit was purchased from Yishan Biotech (Shanghai, China). The polymerase chain reaction (PCR) kit for mRNA detection and miRNA detection were purchased from Takara (Takara Biotechnology, Dalian, China) and Sangon Biotech (Shanghai, China), respectively. Dual-luciferase kit was obtained from Yeasen (Shanghai, China).

### Animals and Treatments

Seven-week-old male C57BL/6J mice and obese (ob/ob) mice with C57BL/6J genetic characteristics were purchased from Jiangsu GemPharmatech Co., Ltd. (Nanjing, China). The mice were kept under suitable temperature and humidity, with a 12 h light-dark cycle. After acclimation to the new environment for a week, the mice were divided into three groups: (1) ND group (n = 7): mice were fed with ND; (2) ob/ob group (n = 7): ob/ob mice were fed with ND; (3) HFD group (n = 7): mice were fed with HFD.

Body weights were recorded each day. The mice were group-housed and provided food and water ad libitum as a recent study described (28). After treatment with HFD or ND for 12 weeks, mice were anesthetized using an intraperitoneal injection of 1% pentobarbital sodium (50 mg/kg), and blood samples and liver tissues were collected.

Experimental protocols were approved by the Animal Care and Use Committee of Fudan University (Shanghai, China). Efforts were made to refine the welfare and reduce the suffering of the experimental mice. The animal studies are reported in compliance with the Animal Research: Reporting of Experiments guidelines (Kilkenny C, 2010).

### Liver Specific GAS5 Overexpression

=AAV8-GAS5 plasmid and AAV8-GFP with virus titre more than 10^13^ vg/ml were constructed by Vigene (Jinan, China). Liver-specific GAS5 overexpression in C57BL/6J mice was established using an injection of AAV8-GAS5 plasmid. Briefly, after a week of acclimatation, seven-week-old male C57BL/6J mice were then divided into four groups (n=7): (1) older ND-fed group (OND), (2) older HFD-fed group (OHFD), (3) AAV8-GAS5 group and (4) AAV8-GFP group. Mice in AAV8-GAS5 group were injected intravenously with a mixture of 7 μl AVV8-GAS5 and 193 μl saline, while that of the AAV8-GFP group were injected with AAV8-GFP. Plasmid was stably expressed in mice 3 weeks after tail vein injection (29). During the 3 weeks, mice in all four group were fed with ND, and then mice of OND group were still fed with ND, while the other three groups were fed with HFD for the next 12 weeks.

### *In Vitro* Models

Two normal human hepatocyte lines, HL-7702 (L-02; CVCL_6926) and QSG-7701 (7701; 3131C0001000200007) were purchased from Yuanchuang Biological Technology Co., Ltd. (Shanghai, China) and complied with the cell STR identification standards established by the International Committee for Cell Identification. Cells were cultured in DMEM containing 10% FBS and maintained in an incubator at 37°C with 5% CO_2_. To establish *in vitro* models, L02 and 7701 cells were treated with a free fatty acid (FFA) mixture (1mM, OA:PA, 2:1 in DMEM with 10% FBS) for 24 h. H89 is a specific PKA inhibitor and forskolin is a cAMP activator (30). L02 and 7701 cells were cultured in DMEM containing 10% FBS and treated with 20 μM H89 or forskolin mixed with DMEM for 1 h before FFA stimulation.

### Plasmid and Small Interfering RNA (siRNA) Transfection *In Vitro*

Full-length GAS5 sequence was cloned into the vector pcDNA3.1 (+) (GenePharma, Shanghai, China). Specific siRNAs for GAS5 and PDE4B as along with a mimic and an inhibitor of miR-26a-5p were designed and synthesised by GenePharma. Oligonucleotide sequences are shown in **Table 1**. Cells were seeded into six-well plates, and the transfection experiments were performed using Lipofectamine 3000, following the manufacturer’s instructions.

**Table 1:** Small interfering RNAs (siRNAs) used in this study.

Name	Sense (5’-3’)	Antisense (5’-3’)
GAS5-1	GCUCUGGAUAGCACCUUAUTT	AUAAGGUGCUAUCCAGAGCTT
GAS5-2	GCAGACCUGUUAUCCUAAATT	UUUAGGAUAACAGGUCUGCTT
GAS5-3	GGACCAGCUUAAUGGUUCUTT	AGAACCAUUAAGCUGGUCCTT
PDE4B-1	GACGCUCAGACACCUAUUATT	UAAUAGGUGUCUGAGCGUCTT
PDE4B-2	CUGCCGAGUUCAUCUAUUATT	UAAUAGAUGAACUCGGCAGTT
PDE4B-3	CGGGAACAGAGAAUGUUUATT	UAAACAUUCUCUGUUCCCGTT
siRNA NC	UUCUCCGAACGUGUCACGUTT	ACGUGACACGUUCGGAGAATT
miR-26a-5p mimic	UUCAAGUAAUCCAGGAUAGGCU	–
miRNA mimic NC	UUGUACUACACAAAAGUACUG	–
miR-26a-5p inhibitor	AGCCUAUCCUGGAUUACUUGAA	–
miRNA inhibitor NC	CAGUACUUUUGUGUAGUACAA	–

### Histological Analysis and Oil Red O Staining

Livers were collected and fixed in 4% paraformaldehyde for more than 24 h. Tissues were embedded with paraffin, and 5 μm sections were sliced for HE and oil red O staining. The oil red O staining was performed as previously described (31).

### Biochemical Analysis of Tissues and Cells

Liver tissues and cell samples were homogenised using PBS or lysed using RIPA lysis buffer. Following this, TG, MDA and SOD levels were detected using the commercial test kits. cAMP levels in the liver tissues and cells were detected using an ELISA kit according to the manufacturer’s protocol.

### RNA Extraction and Quantitative Real-Time PCR (RT-qPCR)

Total RNA was obtained from the liver tissues and cells using an RNA extraction kit and RT-qPCR was performed following the manufacturer’s instructions using a QuantStudio 3 (Thermo Fisher Scientific) thermocycler. β-actin and U6 small nuclear RNA were used for the normalisation of mRNA and miRNA, respectively. Primers used for RT-qPCR in the study are presented in **Table 2**.

**Table 2:** Primers used for qPCR in the study: For mice.

Gene name	Forward (5’-3’)	Reverse (5’-3’)	Gene ID
β-actin	GTGACGTTGACATCCGTAAAGA	GCCGGACTCATCGTACTCC	11461
SREBP-1c	TTGTGGAGCTCAAAGACCTG	TGCAAGAAGCGGATGTAGTC	20787
FAS	GCTGCGGAAACTTCAGGAAAT	AGAGACGTGTCACTCCTGGACTT	14104
ACC1	ATGCGATCTATCCGTCGGTG	AGCAGTTCTGGGAGTTTCGG	104371
ACLY	TTCGTCAAACAGCACTTCC	ATTTGGCTTCTTGGAGGTG	104112
DGAT1	GGAATATCCCCGTGCACAA	CATTTGCTGCTGCCATGTC	13350
CD36	ATGGGCTGTGATCGGAACTG	TTTGCCACGTCATCTGGGTTT	12491
GAS5	CTTGCCTGGACCAGCTTAAT	CAAGCCGACTCTCCATACCT	14455
miR-26a	AAGCTGAGTTCAAGTAATCCAGG	–	387218
PDE4B	GACCGGATACAGGTTCTTCG	CAGTGGATGGACAATGTAGTCA	18578
PGC-1α	TATGGAGTGACATAGAGTGTGCT	GTCGCTACACCACTTCAATCC	19017
CPT-1α	TGGCATCATCACTGGTGTGTT	GTCTAGGGTCCGATTGATCTTTG	25757
For human:			
Gene name	Forward (5’-3’)	Reverse (5’-3’)	Gene ID
β-actin	CTGGAACGGTGAAGGTGACA	AAGGGACTTCCTGTAACAATGCA	60
FAS	GGGATGAACCAGACTGCGTG	TCTGCACTTGGTATTCTGGGT	2194
ACC1	AATGTCCTTCTCCTCCAA	GAGTGAATGAGTTGTCCAA	31
GAS5	AGTAGCCGTCTCTGTGT	CTCCACGAACAGCTTCACAA	60674
miR-26a	TTCAAGTAATCCAGGATAGGCT	–	407015
PDE4B	CTATACCGATCGCATTCAGGTC	CTGTCCATTGCCGATACAATT	5142
PGC-1α	TCTGAGTCTGTATGGAGTGACAT	CCAAGTCGTTCACATCTAGTTCA	10891
CPT-1α	TCCAGTTGGCTTATCGTGGTG	TCCAGAGTCCGATTGATTTTTGC	1374
TFAM	ATAAAGAAGAGATAAGCAGATT	TGCCTATTAAGAGAAAACTAC	7019
NRF-1	GTACAAGAGCATGATCCTGGA	GCTCTTCTGTGCGGACATC	4899

A universal U6 reference forward primer, and universal PCR reverse primer were provided by the miRNA First Strand cDNA Synthesis kit.

### Western Blot

Total protein was extracted from the liver tissues or cell samples using RIPA lysis buffer. Antibodies including Adenosine triphosphate citrate lyase (ACLY, Cat No. 15421-1-AP), acetyl-coenzyme A carboxylase 1 (ACC1, Cat No. 21923-1-AP), fatty acid synthase (FAS, Cat No. 13098-1-AP), GAPDH (Cat No. 60004-1-Ig), peroxisome proliferator-activated receptor γ coactivator-1α (PGC-1α, Cat No. 66369-1-Ig) and cAMP-response element-binding protein (CREB, Cat No. 12208-1-AP) were purchased from Proteinech Group (IL, USA), while phosphodiesterase (PDE) 4B (Cat No. 72096) and phosphorylated CREB (p-CREB, Cat No. 9198) were purchased from Cell Signalling Technology (CST, MA, USA). The dilution of antibodies used in this study were as follows: ACLY (1:1000), ACC1 (1:1000), FAS (1:1000), GAPDH (1:5000), PGC-1α (1:1000), p-CREB (1:500), CREB (1:1000), and PDE4B (1:1000). Western blotting was performed according to the standard protocol and different bands were detected using ECL chromogenic reagent.

### Immunofluorescence

L02 and 7701 cells were seeded on sterile glass coverslips and gently washed with PBS. Samples were fixed with 4% paraformaldehyde for 15 min and rinsed thrice for 5 min each. After 5 min of incubation with 0.2% Triton X, samples were immersed in 5% BSA for 1 h. Blocking buffer was then aspirated and coverslips were incubated with p-CREB (1:100, Cat No. 9198, CST, MA, USA) at room temperature for 2 h. The target antigen was visualised using a fluorescent microscope after incubation with an AlexaFluor488-labeled secondary antibody (Invitrogen, CA, USA) and 4’, 6-diamidino-2-phenylindole (DAPI).

### Luciferase Reporter Assay

The human embryonic kidney (HEK) 293T cell line was purchased from the Cell Bank of Type Culture Collection of the Chinese Academy of Sciences and cells were cultured in DMEM with 10% FBS. The binding sites of has-miR-26a-5p within GAS5 and PDE4B were predicted using ENCORI (https://starbase.sysu.edu.cn). The full-length of GAS5 and fragment of PDE4B 3′-UTR containing the predicted binding sites were amplified using PCR and cloned into a pmirGLO vector (AZENTA, Suzhou, China). The binding sites were mutated to establish the mutant (MUT) model. The reported plasmid and has-miR-26a-5p mimic or inhibitor were co-transfected into HEK 293T. Additionally, a commercial dual-luciferase kit was used to detect the luciferase assay after 36 h of transfection.

### Relative Quantitative Lipidome Analysis of Liver Tissues

Hepatic lipids were extracted with the methyl tert-butyl ether (MTBE) method. Briefly, 30 mg of the liver sample was mixed with 200 μl H_2_O and vortexed for 30 s. Subsequently, 240 μl of precooling methanol was added to the mixture and vortexed for 30 s. Following this, 800 μl of MTBE was added to the mixture, which underwent ultrasound for 20 min at 4°C. The solution was centrifuged at 14000 g for 15 min at 10°C and the upper organic solvent layer was obtained and dried using nitrogen gas. The lipid extracts were re-dissolved in 200 μl 90% isopropanol/acetonitrile and centrifuged at 14000 g for 15 min, and the mass spectra was acquired using Q Exactive Plus system (Thermo Fisher).

### Untargeted Metabolome Analysis of Liver Tissues

Livers were frozen in liquid nitrogen immediately after dissection. At least 80 mg tissues were cut and homogenised with 200 μl of H_2_O and five ceramic beads. Then, 800 of μl methanol/acetonitrile (1:1, v/v) was added to the homogenised solution for metabolite extraction. The mixture was centrifuged for 15 min (14000 g, 4°C) and the supernatant dried in a vacuum centrifuge. For LC-MS analysis, the samples were re-dissolved in 100 μl acetonitrile/water (1:1, v/v) solvent. Samples were separated using an Agilent 1290 Infinity LC ultra-high performance liquid chromatography system (UHPLC, Agilent, CA, USA) and analysed using a Triple TOF 6600 mass spectrometer (AB SCIEX, CA, USA).

### Statistical Analyses

All data are expressed as mean ± standard error of mean. Comparisons between groups were determined using unpair Student’s t test or the Analysis of Variance (ANOVA). Statistical analyses were performed using GraphPad Prism 8 software (GraphPad Software, CA, USA). *P* value < 0.05 indicated significant difference. All data are available from the authors on reasonable request.

## Results

### GAS5 Is Elevated in Ob/Ob Mice, HFD Fed Mice and FFA Stimulated Hepatocytes

**Figure 1:**
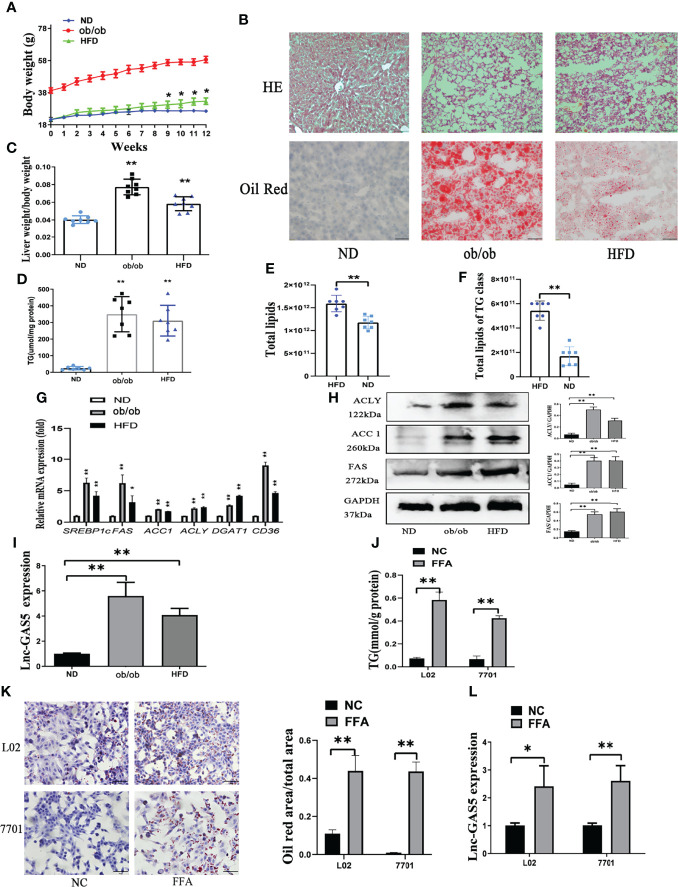
The expression of long non-coding RNA growth arrest specific 5 (GAS5) in *in vivo* and *in vitro* non-alcoholic liver disease (NAFLD) models. The body weight of mice in normal chow diet (ND), obese (ob/ob) and high fat diet (HFD) groups were recorded **(A)**. After 12 weeks of HFD treatment, livers were collected for haematoxylin and eosin staining and oil red staining (Original magnification, ×200) **(B)**. The ratio of liver weight to body weight was calculated **(C)**. Levels of triglycerides (TGs) in the liver tissues were detected using commercial kits **(D)**. Total lipids in HFD and ND fed mice and TG concentration was detected using mass spectrometry **(E, F)**. The gene expression of SREBP-1c, FAS, ACC1, ACLY, DGAT1 and CD36 was detected using quantitative real-time polymerase chain reaction (RT-qPCR) **(G)**. Protein expression of ACLY, ACC1 and FAS was determined using western blotting **(H)**. Expression of GAS5 in ND, ob/ob and HFD mice were detected using RT-qPCR **(I)**. TG levels **(J)**, oil red staining (Original magnification, ×200) **(K)**, and lnc-GAS5 expression **(L)** of cells treated with FFA. Data are expressed as mean ± standard error of mean. *P < 0.05 and **P < 0.01; n = 7 in C-F and n = 3 in G-L.

The body weights of mice in different groups were recorded every week (**Figure 1A**). HFD mice grew significantly heavier than ND mice and there was significant difference from the 9^th^ week. Importantly, HE and oil red O staining showed that ob/ob and HFD mice produced more lipid droplets in the hepatic intracellular vacuoles than the ND mice (**Figure 1B**). Correspondingly, the ratio of liver weight to body weight in the ob/ob and HFD mice was much higher than in the ND group (**Figure 1C**). Notably, the hepatic TG content was increased in the ob/ob and HFD mice (**Figure 1D**). Relative quantitative lipidome analyses of HFD and ND mice were conducted to determine lipid concentrations between the HFD and ND groups (**Figures S1, 2**). HFD fed mice had significantly higher levels of total lipids and TGs compared with that of the ND fed mice (**Figures 1E, F**).

DNL, lipid uptake and lipoprotein secretion contributed to hepatic lipid metabolism. As shown in **Figure 1G**, the expression of key genes associated with DNL, lipid uptake and lipoprotein secretion were significantly increased in ob/ob and HFD fed mice. Consistently, the protein levels of DNL-associated enzymes were also increased in the ob/ob and HFD mice (**Figure 1H**). Therefore, the NAFLD mice models were successfully established, and the expression of GAS5 was upregulated in the ob/ob and HFD mice (**Figure 1I**).

L02 and 7701 cells were used to establish the *in vitro* models. FFA caused an increase in the intracellular TG content (**Figure 1J**), which was evidenced by the increased lipid droplet production in the FFA-treated cells (**Figure 1K**). Moreover, GAS5 expression was higher in FFA treated cells than control cells (**Figure 1L**).

### GAS5 Overexpression Aggravates Lipid Accumulation

**Figure 2:**
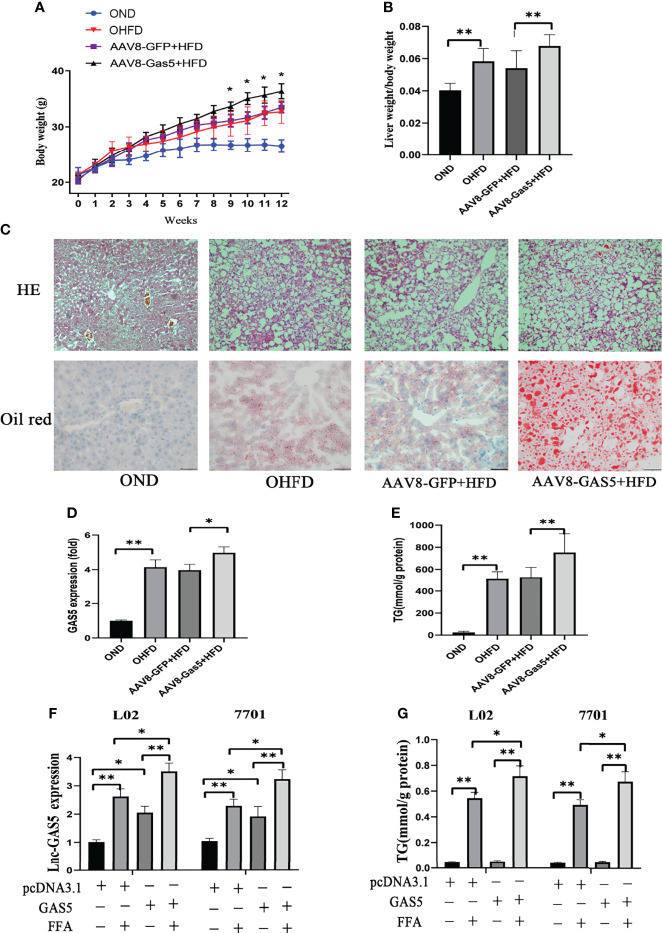
Long non-coding RNA growth arrest specific 5 (GAS5) overexpression aggregates hepatic lipid accumulation. After mice were injected with AAV8-GAS5 or AAV8-GFP for 21 days, they were fed with high fat diet (HFD) or normal chow diet (ND). The body weight of mice in OND, OHFD, AAV8-GFP plus HFD and AAV8-GAS5 plus HFD group was recorded every week **(A)**, data are expressed as mean ± standard error of mean (SEM), *P < 0.05, n = 7. The ratio of liver weight to body weight in different groups were calculated **(B)**, data are expressed as mean ± SEM, **P<0.01, n = 7. The expression of GAS5 in different groups **(C)**, data are expressed as mean ± standard error of mean (SEM), *P<0.05, **P<0.01, n = 3. Haematoxylin and eosin staining and oil red staining of liver tissues in different groups (Original magnification, ×200) **(D)**. The detection of hepatic triglycerides (TGs) in different groups **(E)**, data are expressed as mean ± SEM, **P<0.01, n = 7. Cells were transfected with pcDNA3.1-GAS5 (described as GAS5) or pcDNA3.1 vector and treated with free fatty acid. The quantitative real-time polymerase chain reaction analysis of GAS5 in different groups **(F)**, and the TG detection in different groups **(G)**; data are expressed as mean ± SEM, *P < 0.05, **P < 0.01, n = 3.

Compared with the AAV8-GFP plus HFD groups, mice in the AAV8-GAS5 plus HFD group developed a significantly higher body weight and liver/body weight ratio (**Figures 2A, B**). Notably, increased lipid droplet accumulation in the hepatic intracellular vacuoles was observed in the GAS5 overexpressed group (**Figure 2C**). AVV8-GAS5 significantly increased the expression of GAS5 in liver tissues (**Figure 2D**). Changes in the biochemical parameters were observed, with increased hepatic TG levels in GAS5 overexpressed mice (**Figure 2E**).

Simultaneously, this study confirms that the expression of GAS5 in cells was upregulated with pcDNA-GAS5 transfection (described as GAS5 in Figures) (**Figure 2F**). Furthermore, GAS5 overexpression resulted in increased TG synthesis in FFA-stimulated cells (**Figure 2G**). Therefore, GAS5 overexpression is closely associated with hepatic and hepatocellular lipid accumulation.

### GAS5 Knockdown Alleviates Hepatocellular DNL Lipogenesis

**Figure 3:**
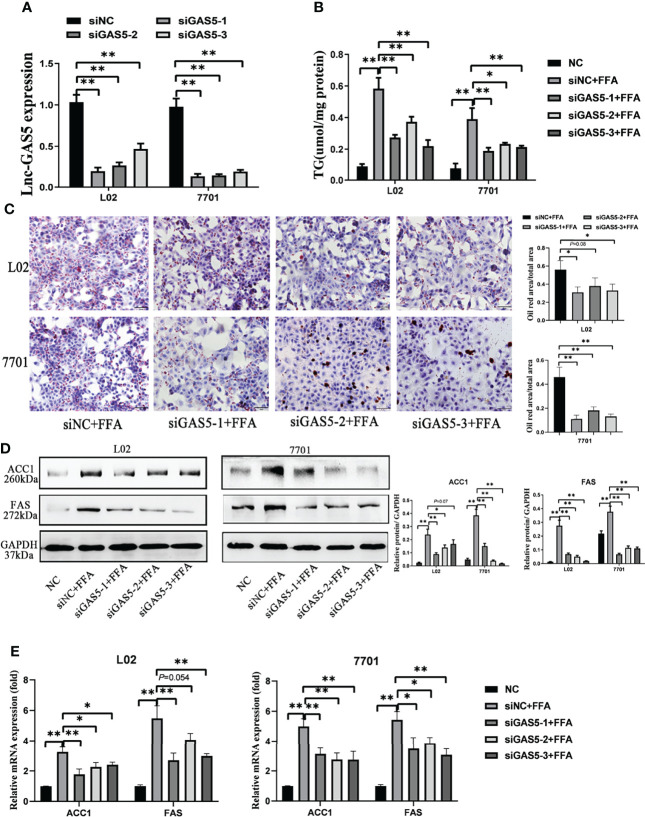
Long non-coding RNA growth arrest specific 5 (GAS5) knockdown mitigates cellular *de novo* lipogenesis. After cells were transfected with three synthetic small interfering RNAs, the expression of lnc-GAS5 were determined using quantitative real-time polymerase chain reaction analysis (RT-qPCR) **(A)** and lipid accumulation in cells were assessed *via* oil red staining (Original magnification, ×200) **(B)**. The cellular triglyceride levels were detected using commercial kits **(C)**, and the protein and mRNA levels of ACC1 and FAS were measured *via* western blot and RT-qPCR, respectively **(D, E)**. Data are expressed as mean ± standard error of mean. *P<0.05, and **P<0.01, n = 3.

All three synthetic siRNA for GAS5 (siGAS5) significantly downregulated the intracellular GAS5 levels (**Figure 3A**). TG contents in the cells were also reduced after GAS5 knockdown (**Figure 3B**). Correspondingly, decreased lipid droplets were produced in the cells (**Figure 3C**). Moreover, the protein expression of DNL enzymes, ACC1 and FAS was downregulated in FFA-treated cells after GAS5 suppression (**Figure 3D**). Similar results were observed in the mRNA expression of ACC1 and FAS (**Figure 3E**). Considering that siGAS5-1 had the strongest inhibitory effect on GAS5 expression and had an obvious effect on inhibiting lipid droplet formation and reducing the expression of ACC1 and FAS in L02 and 7701 cells, we selected siGAS5-1 for subsequent experiments and described it as siGAS5.

### GAS5 Knockdown Preserves Mitochondrial Function

**Figure 4:**
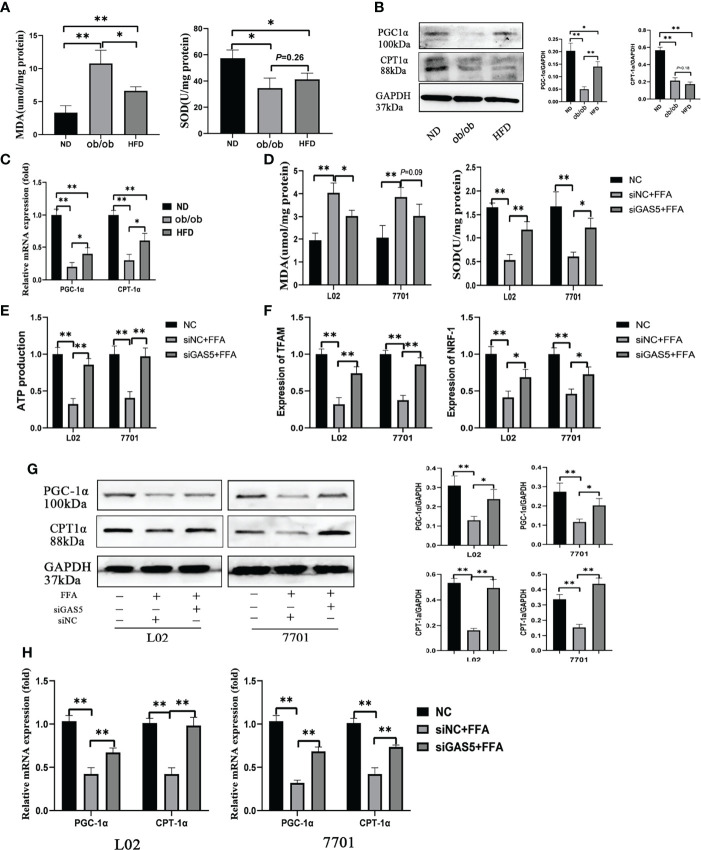
Long non-coding RNA growth arrest specific 5 (GAS5) regulates mitochondrial function. The detection of hepatic malondialdehyde (MDA) and superoxide dismutase (SOD) levels in different groups of mice **(A)**. The protein and mRNA expression of PGC-1α and CPT-1α **(B, C)**. Cells were transfected with small interfering GAS5-1 (described as siGAS5 in subsequent experiments) and then treated with free fatty acid. The detection of cellular MDA and SOD levels **(D)**. The detection of ATP production in cells of FFA, siNC plus FFA and siGAS5 plus FFA groups **(E)**. The expression of mRNA levels of mitochondrial biogenesis including TFAM and NRF-1 in cells of FFA, siNC plus FFA and siGAS5 plus FFA groups **(F)**. The protein and mRNA expression of PGC-1α and CPT-1α in different groups of cells **(G, H)**. Data are expressed as mean ± standard error of mean. *P < 0.05 and **P < 0.01, n = 3.

MDA and SOD are oxidative stress markers, and the increased levels of MDA and decreased levels of SOD indicate that the mitochondrial function is suppressed in the ob/ob and HFD treated mice (**Figure 4A**). PGC-1α and CPT-1α play important roles in fatty acid oxidation and are key indicators of mitochondrial function, which were reduced in the ob/ob and HFD-fed mice (**Figures 4B, C**).

In L02 and 7701 cells, the levels of MDA were upregulated and SOD were downregulated in FFA-stimulated cells and the changes in MDA and SOD were reversed on GAS5 knockdown (**Figure 4D**). FFA treatment markedly decreased ATP production, but GAS5 knockdown could upregulate the ATP production in FFA-stimulated cells (**Figure 4E**). Furthermore, GAS5 knockdown increased the mRNA expression of TFAM and NRF-1 (**Figure 4F**). In **Figures 4G, H**, GAS5 knockdown helped cells maintain the levels of PGC-1α and CPT-1α. Hence, GAS5 knockdown can protect the mitochondrial function.

### GAS5 Modulates PDE4B Expression by Sponging With MiR-26a-5p

**Figure 5:**
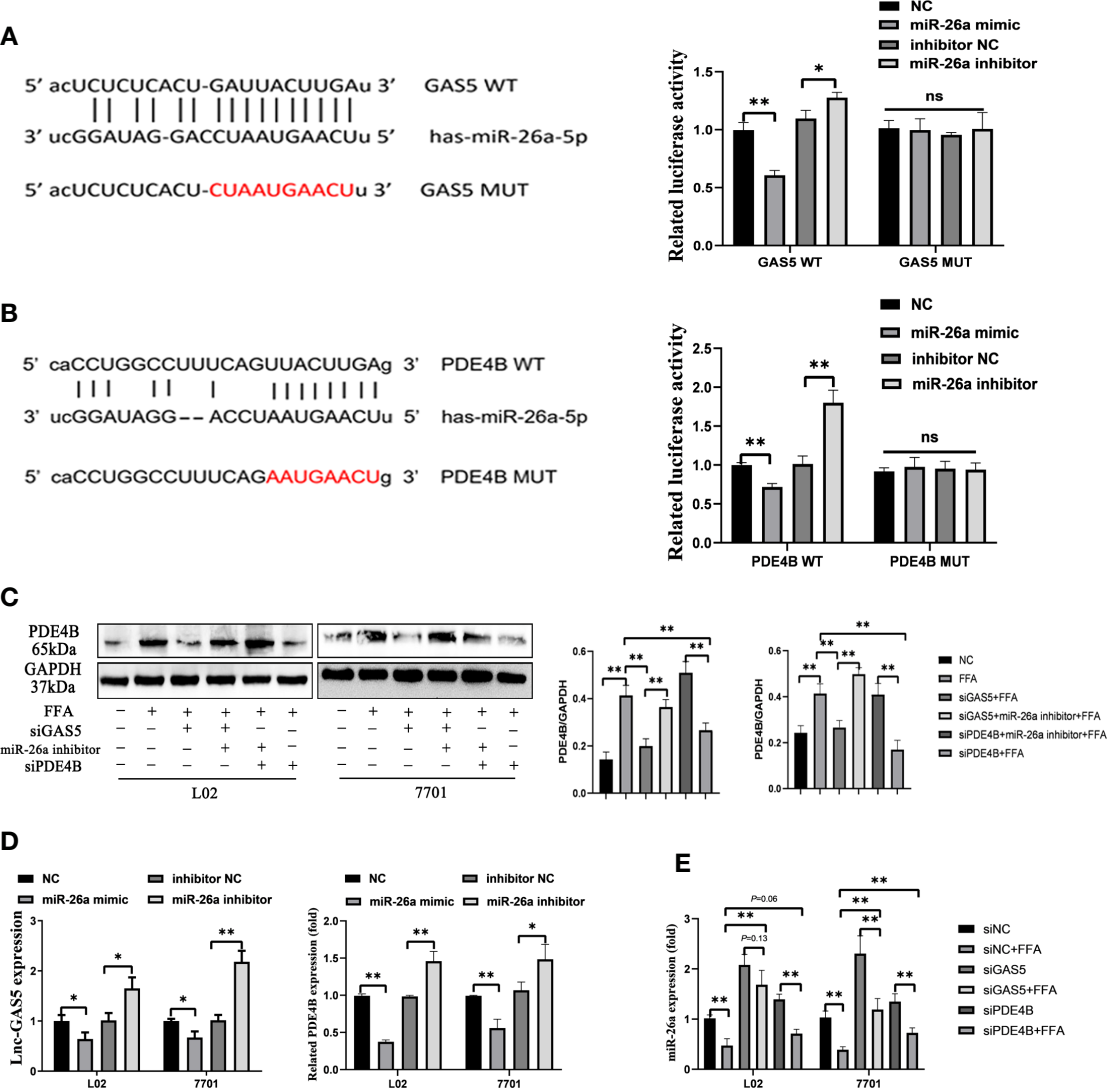
Long non-coding RNA growth arrest specific 5 (GAS5) regulates phosphodiesterase-4D (PDE4B) by sponging miR-26a-5p. Bioinformatic predictions of the miR-26a-5p targeting GAS5 3′UTR through the binding sequence, and dual luciferase reporter detection of GAS5 and miR-26a-5p **(A)**. Bioinformatic predictions of the miR-26a-5p targeting PDE4B 3′UTR through the binding sequence, and dual luciferase reporter detection of PDE4B and miR-26a-5p **(B)**. The protein expression of PDE4B in different groups **(C)**. The expression of GAS5 and PDE4B of cells transfected with a miR-26a mimic or inhibitor **(D)**. The expression of miR-26a-5p in cells with GAS5 knockdown or PDE4B knockdown **(E)**. Data are expressed as mean ± standard error of mean. *P < 0.05 and **P < 0.01, n = 3. ns, not significant.

Sequencing analysis showed the potential binding sites between GAS5 and miR-26a-5p. Subsequently, the luciferase reporters were constructed, containing the wild-type GAS5 (GAS5 WT) or mutant GAS5 (GAS5 MUT). Compared with miRNA negative control (NC) or inhibitor NC, miR-26a-5p mimic or inhibitor suppressed or enhanced the luciferase reporter activities of GAS5 WT, but not those of GAS5 MUT (**Figure 5A**). Potential binding sites were also predicted between PDE4B and miR-26a-5p and further proved by luciferase reporters (**Figure 5B**). Therefore, GAS5 and PDE4B mRNA are physically associated with miR-26a-5p *via* these sites.

All three siRNA for PDE4B significantly reduced the expression of PDE4B in cells, and we chose siPDE4B-2 for subsequent experiments and described it as siPDE4B (**Figure S3**). GAS5 knockdown decreased PDE4B protein expression, whereas PDE4B expression was increased on miR-26a-5p inhibition. Decreased miR-26a-5p could promote PDE4B expression even when cells were treated with siPDE4B (**Figure 5C**). The overexpression of miR-26a-5p suppressed the levels of GAS5, while decreased miR-26a-5p upregulated the expression of GAS5. As a downstream molecule of miR-26a-5p, there exists an inverse correlation between PDE4B and miR-26a-5p (**Figure 5D**). GAS5 knockdown promoted the expression of miR-26a-5p in FFA-treated cells. Moreover, the decreased level of PDE4B also upregulated miR-26a-5p expression (**Figure 5E**). Hence, GAS5 sponges with miR-26a-5p and modulates PDE4B expression.

### GAS5/MiR-26a-5p/PDE4B Axis Mediates FFA-Induced Hepatocellular Lipid Metabolism

**Figure 6:**
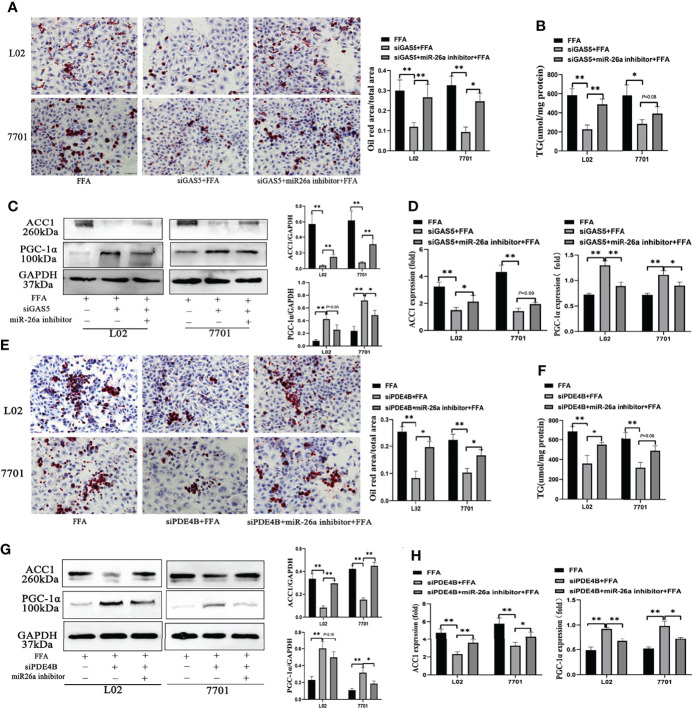
The regulation of the long non-coding RNA growth arrest specific 5 (GAS5)/miR-26a-5p/phosphodiesterase-4D (PDE4B) axis in lipid metabolism. Cells were transfected with small interfering (si)GAS5 or siGAS5 plus miR-26a-5p inhibitor and stained with oil red (Original magnification, ×200) **(A)**. Triglyceride (TG) levels were detected using a commercial kit **(B)**, and the protein and mRNA expression of ACC1 and PGC-1α was detected *via* western blot and quantitative real-time polymerase chain reaction analysis (RT-qPCR), respectively **(C, D)**. Cells were stained with oil red after transfection with siPDE4B or siPDE4B plus a miR-26a-5p inhibitor, (Original magnification, ×400) **(E)**, TG levels detection **(F)**, and the protein and mRNA expression of ACC1 and PGC-1α was detected *via* western blot and RT-qPCR, respectively **(G, H)**. Data are expressed as mean ± standard error of mean. *P < 0.05 and **P < 0.01, n = 3.

When cells were transfected with siGAS5 and miR-26a-5p inhibitor, lipid accumulation and intracellular TG levels were increased compared to those treated with siGAS5 plus FFA (**Figures 6A, B**). Western blot and qRT-PCR results showed that GAS5 knockdown inhibited ACC1 expression and promoted PGC-1α expression, whereas these characteristics could be restricted on co-transfection with miR-26a-5p inhibitor (**Figures 6C, D**).

FFA-stimulated cellular lipid accumulation decreased with PDE4B downregulation, whereas it was increased with a miR-26a-5p inhibitor co-transfection (**Figure 6E**). Consistently, intracellular TG levels in PDE4B knockdown cells could be upregulated by a miR-26a-5p inhibitor (**Figure 6F**). In **Figure 6G**, the protein levels of ACC1 decreased when PDE4B was downregulated and this could be reversed by a miR-26a-5p inhibitor. As a protective molecule, changes in PGC-1α were contrasting to those in ACC1. Additionally, ACC1 and PGC-1α mRNA expression detection *via* RT-qPCR showed similar results (**Figure 6H**). Therefore, GAS5 regulates hepatocellular lipid accumulation *via* the miR-26a-5p/PDE4B axis.

### Lnc-GAS5 Modulates cAMP/CREB Pathway

As a hepatic manifestation of metabolic syndromes, changes in hepatocellular metabolites can aid in elucidating the regulatory processes of NAFLD. The hepatic untargeted metabolomic analysis of HFD and ND mice were performed to demarcate the HFD and ND groups (**Figures S4, 5**). The concentration of 144 metabolites changed on comparing the HFD-fed and ND-fed mice (**Figure S6A**), and different pattern of lipid and lipid like metabolites were shown in **Figure S6B**. The 20 most enriched Kyoto Encyclopaedia of Genes and Genomes pathways based on metabolite variations are shown in **Figure S6C**. Given the close association between PDE4B and cAMP, the changes and roles of the cAMP pathway were analysed in this study’s models.

**Figure 7:**
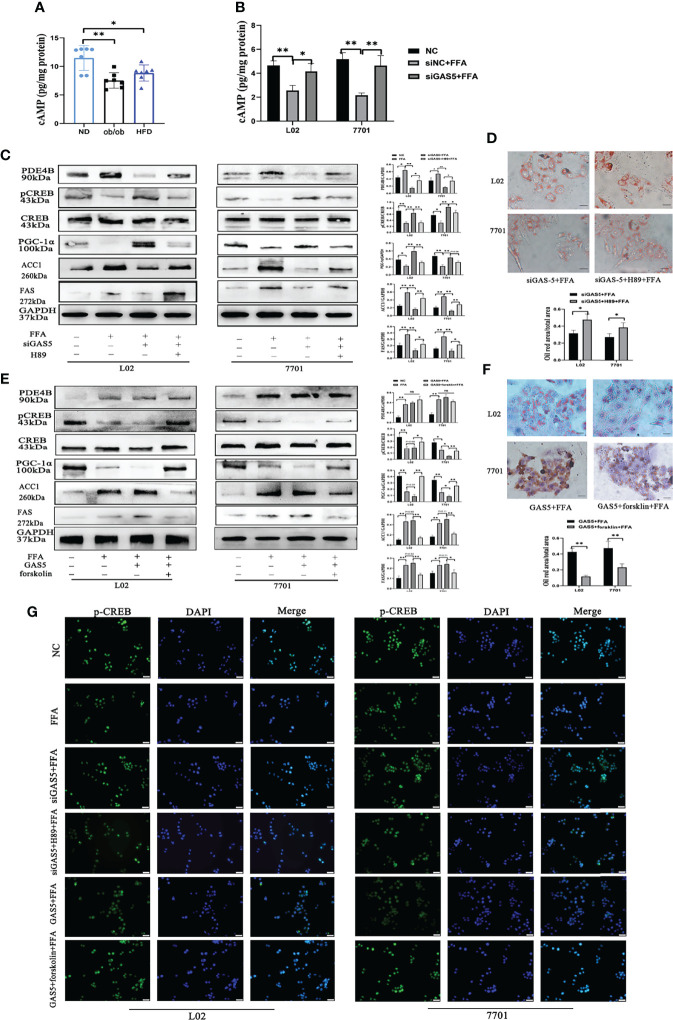
Long non-coding RNA growth arrest specific 5 (GAS5) regulates cAMP/CREB pathway through phosphodiesterase-4B (PDE4B). The cAMP levels in liver tissues **(A)** and cells treated with free fatty acid (FFA) or siGAS5 plus FFA **(B)**. Protein expression of PDE4B, p-CREB, CREB, PGC-1α, ACC1 and FAS in cells treated with FFA after transfection with siGAS5 or siGAS5 plus H89 **(C)**. Oil red staining of cells transfected with siGAS5 or siGAS5 plus H89 followed by FFA treatment (Original magnification, ×400) **(D)**. Protein expression of PDE4B, p-CREB, CREB, PGC-1α, ACC1 and FAS in cells treated with FFA after transfection with GAS5 or GAS5 plus forskolin **(E)**. Oil red staining of cells transfected with GAS5 or GAS5 plus forskolin followed by FFA treatment (Original magnification, ×400) **(F)**. Immunofluorescence of p-CREB in cells with different treatment **(G)**. Data are expressed as mean ± standard error of mean. *P < 0.05 and **P < 0.01, n = 3.

The variation of metabolites involved in the cAMP pathway were collected and significant differences between the HFD and ND groups were observed. The expression of 3-hydroxybutyrate and acetylcholine was downregulated in HFD-fed mice, whereas the hydrolysates of cAMP, adenosine 5’-monophosphate and adenosine were increased in HFD-fed mice (**Figure S6D**). Furthermore, HFD feeding decreased the levels of hepatic cAMP (**Figure 7A**). Similar results were detected in FFA-treated cells, whereas GAS5 knockdown increased the cAMP accumulation (**Figure 7B**). FFA treatment increased the expression of PDE4B and suppressed the expression of p-CREB and PGC-1α. Knockdown of GAS5 promoted CREB phosphorylation and reversed PGC-1α alteration. On the contrary, H89 dephosphorylated CREB and reduced PGC-1α expression (**Figure 7C**). Furthermore, H89 aggravated the accumulation of lipids in FFA-stimulated cells compared with those transfected with siGAS5 (**Figure 6D**). Although forskolin did not affect PDE4B expression, it notably promoted CREB phosphorylation and thereby suppressed ACC1 and FAS expression and increased PGC-1α expression (**Figure 7E**). Forskolin treatment reduced lipid droplet accumulation even when GAS5 was overexpressed (**Figure 7F**). Integrating the effects of H89 and forskolin on lipid accumulation, the cAMP/CREB pathway was involved in the regulation of GAS5 in hepatocellular lipid metabolism, which was confirmed by the p-CREB immunofluorescence in cells with different treatments. As shown in **Figure 7G**, p-CREB expression increased with siGAS5 transfection and forskolin treatment, but decreased with GAS5 overexpression and H89 treatment in cells, particularly in the nuclei.

## Discussion

The present study demonstrated that the expression of GAS5 was upregulated in fatty liver models *in vitro* and *in vivo*. Additionally, the function of the GAS5/miR-26a-5p/PDE4B and cAMP/CREB pathway in the regulation of hepatic lipid metabolism was investigated.

With various studies focusing on new targets for NAFLD diagnosis and treatment, lncRNAs has been reported as an increasingly promising candidate (32). Given its increased expression in patients with NAFLD (20), GAS5 could have a role in regulating hepatic lipid metabolism. GAS5 was reported to have anti-tumour effects in many kinds of human tumours, however, Tao et al. demonstrated that GAS5 could be a proto-oncogene in HCC (33). That may partly be explained by the high tissue-specific feature of lncRNA, and therefore, the function of lncRNA in different tissues may be different (34). The single nucleotide polymorphism (SNP) detection and more *in vivo* studies will help us better understand the biological functions of lncRNAs (35, 36). This study confirmed that the expression of GAS5 was increased in leptin knockout mice, HFD fed mice and FFA stimulated cells. On elucidating the association between GAS5 and hepatic lipid metabolism, increased hepatic DNL was found to be a central metabolic indicator for NAFLD (6, 37, 38). When energy storage is sufficient, dietary lipids, carbohydrates and proteins act as substrates for DNL (39). The actions of regulatory enzymes, including ACLY, ACC and FAS, have been reported to play a role in the regulation of hepatic fatty acid synthesis (40–42). Increasing DNL is closely associated with iron overload and hepatic inflammation through bone morphogenetic protein (BMP6)/Sma- and Mad-related proteins (SMAD) pathway in NAFLD (43). A recent study showed that increased DNL in mice could reduce the expression of HMG-CoA reductase protein and the excretion of bile acids, and facilitated cholesterol uptake. In addition, DNL also led to the secretion of very low-density lipoproteintriglyceride, which was closely associated with hypertriglyceridemia (44). In NAFLD mice models, SREBP-1c, ACLY, ACC1, FAS, DGAT1 and CD36 were upregulated, with significant hepatic TG accumulation in the pathological sections. Lipidome detection is a novel method to study the pathogenesis of NAFLD or alcohol-induced fatty liver disease (45, 46), which detected both quality and quantity changes in lipids in the mice tissues. Liver-specific GAS5 overexpression in mice accelerated weight gain and TG accumulation, gradually developing severe hepatic steatosis and hepatocellular ballooning. Correspondingly, GAS5 knockdown alleviates the cellular lipid accumulation and downregulates DNL marker expressions, demonstrating that GAS5 is closely associated with the regulation of hepatic DNL.

LncRNAs regulate the expression of target genes by competing for shared microRNAs with mRNAs, which are known as ceRNAs (47). Bioinformatic analyses and luciferase reporter assay showed that GAS5 physically interacted with microRNA-26a-5p (miR-26a-5p) through their binding sites. The interaction between GAS5 and miR-26a-5p has been well demonstrated in this study, with GAS5 sponging with miR-26a-5p; furthermore, GAS5 knockdown upregulates miR-26a-5p (23). However, increased levels of miR-26a have been demonstrated to mitigate NAFLD *in vivo* and *in vitro (* 23, 48, 49). Ding et al. found that miR-26a could downregulate CD36, and thereby reducing the steatosis in HepG2 cells (50). Micewith miR-26a overexpression had significant lower liver weight and TG contents compared with that of the normal control mice (49). Conversely, overexpressed GAS5 notably reduced the expression of miR-26a-5p.

Recently, metabolomic analyses have provided novel insights into the pathophysiology of NAFLD (51, 52). Through the detection of hepatic metabolites, the protective cAMP response pathway was highlighted from the analysis of the KEGG signalling pathways. Known as a second messenger, cAMP can be synthesized by adenylyl cyclase from adenosine triphosphate and initiate downstream molecular interactions (53). In HFD-fed mice or FFA-treated cells, the expression of PDE4B was upregulated. Moreover, PDE4B, which is highly expressed in the liver, selectively hydrolyses cAMP (27). Low levels of cAMP lead to the reduction of mitochondrial function and oxygen consumption, thus promoting NAFLD and insulin resistance (54). Conversely, GAS5 knockdown elevated cAMP, which could be attributed to PDE4B inhibition. Increased cAMP phosphorylates CREB by activating protein kinase A and eventually promoting the induction of peroxisome proliferator-activated receptor (PPAR)-γ coactivator 1α (PGC-1α) (55). However, these changes could be reduced when GAS5 was upregulated. Reduced p-CREB expression exacerbates HFD-induced hepatic steatosis by downregulating PPARα (56), whereas activated p-CREB reduces PPAR-γ expression to regulate the hepatic DNL (57), which was confirmed by the downregulation of DNL-related ACC1 and FAS in GAS5 knockdown cells. However, GAS5 knockdown cells with H89 pre-treatment suppressed PGC-1α expression and increased ACC1 and FAS expression. Additionally, H89 reduced the expression of p-CREB by inhibiting the activity of PKA, and subsequently increasing the expression of SREBP-1c, ultimately leading to DNL (58). Conversely, forskolin pre-treatment increased the level of cAMP, upregulated the expression of PGC-1α and reduced the levels of ACC1 and FAS, without affecting PDE4B. Therefore, GAS5 regulates hepatic lipid metabolism *via* the cAMP response signalling pathway, at least partially.

A recent study identified GAS5 as a mitochondria-associated lncRNA, sustaining homeostasis by regulating mitochondrial metabolic enzymes in physiology and cancer (59). Mitochondria control energy metabolism in cells and pathways involved in the regulation of energy metabolism at least partially dependent on mitochondrial function (60). The PGC1 family, including PGC-1α, PGC-1β and PGC related coactivator (61), regulate the mitochondrial biogenesis by interacting with transcription factors or nuclear receptors (62). The expression of PGC-1α was downregulated in HFD treatment or FFA stimulation in this study, and reduced PGC-1α fails to maintain the content of mitochondrial-associated proteins (63). Moreover, GAS5 knockdown by siRNA significantly upregulated the levels of PGC-1α. Increased PGC-1α could promote the levels of lipin 1, subsequently activating PPARα to improve the mitochondrial fatty acid oxidation capacity and suppress DNL and lipid secretion (64). Carnitine palmitoyltransferase-1α (CPT-1α) is abundant in the liver and plays an important role in mitochondrial oxidation by converting fatty acyl-CoA to fatty acylcarnitine (65). In this study, impaired mitochondrial function after HFD or FFA treatment was indicated with increased MDA levels and decreased SOD levels. Consistent with a previous study, the increased expression of PGC-1α and CPT-1α ameliorated NAFLD by promoting mitochondrial function (31), with GAS5 knockdown increasing the expression of PGC-1α and CPT-1α. Furthermore, the overexpression of GAS5 suppresses the expression of PGC-1α and CPT-1α and aggravates hepatic lipid accumulation and liver damage.

**Figure 8:**
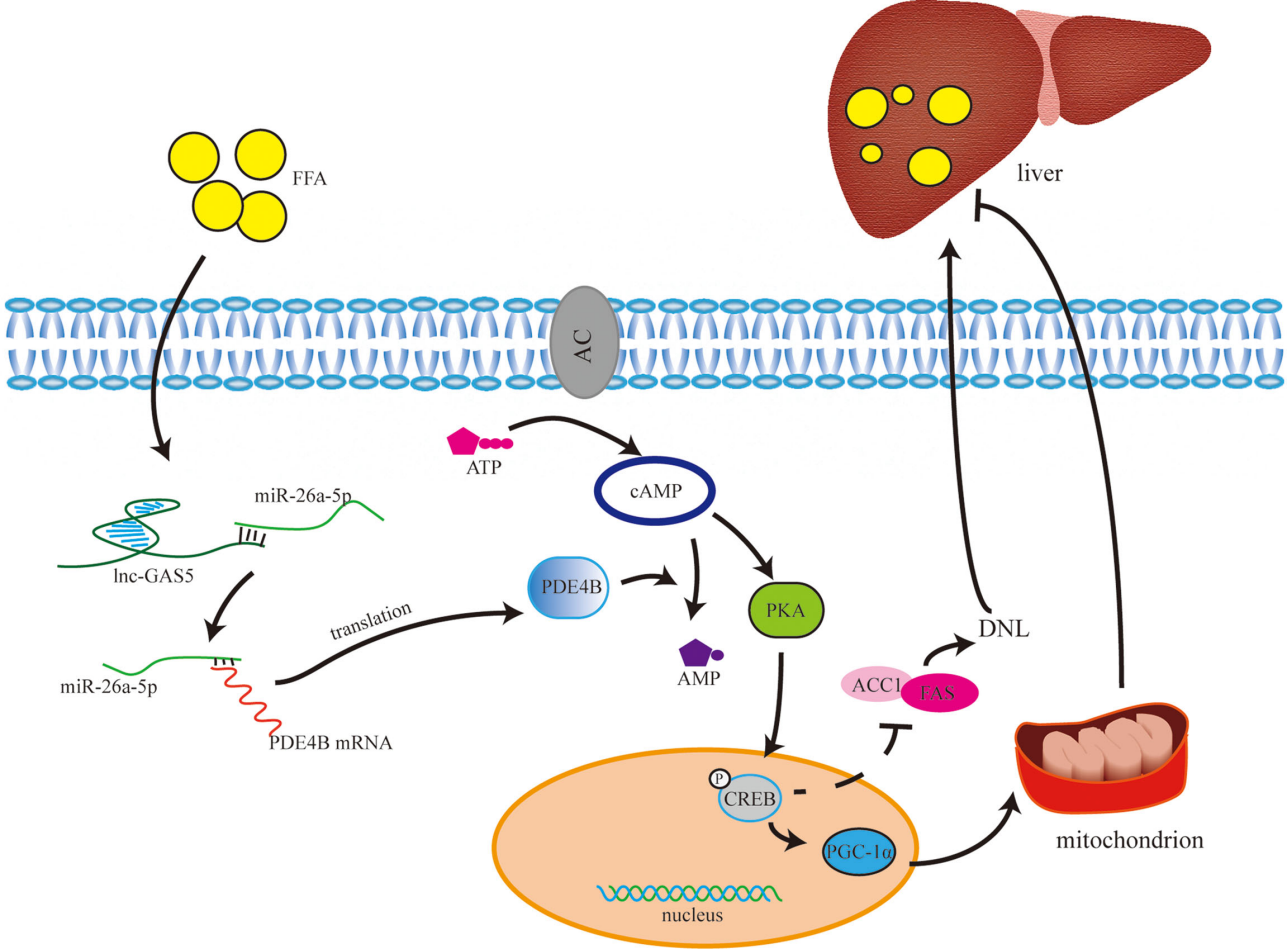
The underlying mechanism of long non-coding RNA growth arrest specific 5 (GAS5) in regulating lipid metabolism. Under normal physiological conditions, cAMP is synthesised from ATP with the catalysis of adenylate cyclase. cAMP activates PKA to phosphorylate CREB, and phosphorylated CREB (p-CREB) upregulates PGC-1α and downregulates ACC1 and FAS expression. Therefore, through controlling the *de novo* lipogenesis (DNL) and mitochondrial function, high level of cAMP may reduce lipid accumulation in liver. cAMP is hydrolysed into AMP by phosphodiesterase, especially phosphodiesterase-4B in liver. GAS5 is upregulated in hepatocytes with high-fat treatment *via* the miR-26a-5p/PDE4B axis, which consumes cAMP, thereby promoting fat accumulation in the liver.

In conclusion, the expression of GAS5 was increased in *in vivo* and *in vitro* fatty liver models. Moreover, GAS5 sponges with miR-26a-5p to upregulate PDE4B expression, thus modulating hepatic lipid metabolism *via* the cAMP/CREB pathway (**Figure 8**). This study provides a novel therapeutic strategy for downregulating GAS5 levels in fatty liver treatment.

## Data Availability Statement

The original contributions presented in the study are included in the article/**Supplementary Material**. Further inquiries can be directed to the corresponding authors.

## Ethics Statement

The animal study was reviewed and approved by Animal Care and Use Committee of Fudan University.

## Author Contributions

XF, MJ, and SX contributed to conception and design of the study. YW and ZL analyzed the data. SX and XF wrote the first draft of the manuscript. MJ and QH wrote sections of the manuscript. All authors contributed to manuscript revision, read, and approved the submitted version.

## Funding

The study was supported by Shanghai Science and Technology Committee (No. 15411969800) and Construction Project of Backup Discipline Platform of Jinshan Hospital Affiliated to Fudan University (No. HBXK-2021-2).

## Conflict of Interest

The authors declare that the research was conducted in the absence of any commercial or financial relationships that could be construed as a potential conflict of interest.

## Publisher’s Note

All claims expressed in this article are solely those of the authors and do not necessarily represent those of their affiliated organizations, or those of the publisher, the editors and the reviewers. Any product that may be evaluated in this article, or claim that may be made by its manufacturer, is not guaranteed or endorsed by the publisher.

## References

[1] Younossi ZM Koenig AB Abdelatif D Fazel Y Henry L Wymer M. Global Epidemiology of Nonalcoholic Fatty Liver Disease-Meta-Analytic Assessment of Prevalence, Incidence, and Outcomes. Hepatology (2016) 64(1):73–84. doi: 10.1002/hep.28431 26707365

[2] Younossi Z Tacke F Arrese M Chander Sharma B Mostafa I Bugianesi E. Global Perspectives on Nonalcoholic Fatty Liver Disease and Nonalcoholic Steatohepatitis. Hepatology (2019) 69(6):2672–82. doi: 10.1002/hep.30251 30179269

[3] Farrell GC Larter CZ. Nonalcoholic Fatty Liver Disease: From Steatosis to Cirrhosis. Hepatology (2006) 43(2 Suppl 1):S99–S112. doi: 10.1002/hep.20973 16447287

[4] Starley BQ Calcagno CJ Harrison SA. Nonalcoholic Fatty Liver Disease and Hepatocellular Carcinoma: A Weighty Connection. Hepatology (2010) 51(5):1820–32. doi: 10.1002/hep.23594 20432259

[5] Hall Z Bond NJ Ashmore T Sanders F Ament Z Wang X. Lipid Zonation and Phospholipid Remodeling in Nonalcoholic Fatty Liver Disease. Hepatology (2017) 65(4):1165–80. doi: 10.1002/hep.28953 PMC5396354 27863448

[6] Lambert JE Ramos-Roman MA Browning JD Parks EJ. Increased *De Novo* Lipogenesis is a Distinct Characteristic of Individuals With Nonalcoholic Fatty Liver Disease. Gastroenterology (2014) 146(3):726–35. doi: 10.1053/j.gastro.2013.11.049 PMC6276362 24316260

[7] Softic S Cohen DE Kahn CR. Role of Dietary Fructose and Hepatic *De Novo* Lipogenesis in Fatty Liver Disease. Dig Dis Sci (2016) 61(5):1282–93. doi: 10.1007/s10620-016-4054-0 PMC4838515 26856717

[8] Begriche K Igoudjil A Pessayre D Fromenty B. Mitochondrial Dysfunction in NASH: Causes, Consequences and Possible Means to Prevent it. Mitochondrion (2006) 6(1):1–28. doi: 10.1016/j.mito.2005.10.004 16406828

[9] Paradies G Paradies V Ruggiero FM Petrosillo G. Oxidative Stress, Cardiolipin and Mitochondrial Dysfunction in Nonalcoholic Fatty Liver Disease. World J Gastroenterol (2014) 20(39):14205–18. doi: 10.3748/wjg.v20.i39.14205 PMC4202349 25339807

[10] Iwabu M Yamauchi T Okada-Iwabu M Sato K Nakagawa T Funata M. Adiponectin and AdipoR1 Regulate PGC-1alpha and Mitochondria by Ca(2+) and AMPK/Sirt1. Nature (2010) 464(7293):1313–9. doi: 10.1038/nature08991 20357764

[11] Puigserver P Wu Z Park CW Graves R Wright M Spiegelman BM. A Cold-Inducible Coactivator of Nuclear Receptors Linked to Adaptive Thermogenesis. Cell (1998) 92(6):829–39. doi: 10.1016/s0092-8674(00)81410-5 9529258

[12] Leone TC Lehman JJ Finck BN Schaeffer PJ Wende AR Boudina S. PGC-1alpha Deficiency Causes Multi-System Energy Metabolic Derangements: Muscle Dysfunction, Abnormal Weight Control and Hepatic Steatosis. PLoS Biol (2005) 3(4):e101. doi: 10.1371/journal.pbio.0030101 15760270 PMC1064854

[13] Hombach S Kretz M. Non-Coding RNAs: Classification, Biology and Functioning. Adv Exp Med Biol (2016) 937:3–17. doi: 10.1007/978-3-319-42059-2_1 27573892

[14] Ma M Duan R Shen L Liu M Ji Y Zhou H. The lncRNA Gm15622 Stimulates SREBP-1c Expression and Hepatic Lipid Accumulation by Sponging the miR-742-3p in Mice. J Lipid Res (2020) 61(7):1052–64. doi: 10.1194/jlr.RA120000664 PMC7328049 32229588

[15] Coccia EM Cicala C Charlesworth A Ciccarelli C Rossi GB Philipson L. Regulation and Expression of a Growth Arrest-Specific Gene (Gas5) During Growth, Differentiation, and Development. Mol Cell Biol (1992) 12(8):3514–21. doi: 10.1128/mcb.12.8.3514-3521.1992 PMC364604 1630459

[16] Smith CM Steitz JA. Classification of Gas5 as a Multi-Small-Nucleolar-RNA (snoRNA) Host Gene and a Member of the 5'-Terminal Oligopyrimidine Gene Family Reveals Common Features of snoRNA Host Genes. Mol Cell Biol (1998) 18(12):6897–909. doi: 10.1128/MCB.18.12.6897 PMC109273 9819378

[17] Gasic V Stankovic B Zukic B Janic D Dokmanovic L Krstovski N. Expression Pattern of Long Non-Coding RNA Growth Arrest-Specific 5 in the Remission Induction Therapy in Childhood Acute Lymphoblastic Leukemia. J Med Biochem (2019) 38(3):292–8. doi: 10.2478/jomb-2018-0038 PMC6534956 31156339

[18] Mourtada-Maarabouni M Hasan AM Farzaneh F Williams GT. Inhibition of Human T-Cell Proliferation by Mammalian Target of Rapamycin (mTOR) Antagonists Requires Noncoding RNA Growth-Arrest-Specific Transcript 5 (GAS5). Mol Pharmacol (2010) 78(1):19–28. doi: 10.1124/mol.110.064055 20421347 PMC2912054

[19] Yu F Zheng J Mao Y Dong P Lu Z Li G. Long Non-Coding RNA Growth Arrest-Specific Transcript 5 (GAS5) Inhibits Liver Fibrogenesis Through a Mechanism of Competing Endogenous RNA. J Biol Chem (2015) 290(47):28286–98. doi: 10.1074/jbc.M115.683813 PMC4653684 26446789

[20] Han MH Lee JH Kim G Lee E Lee YR Jang SY. Expression of the Long Noncoding RNA GAS5 Correlates With Liver Fibrosis in Patients With Nonalcoholic Fatty Liver Disease. Genes (Basel) (2020) 11(5):545. doi: 10.3390/genes11050545 PMC7291058 32413995

[21] Alshehri AS El-Kott AF El-Kenawy AE Khalifa HS AlRamlawy AM. Cadmium Chloride Induces non-Alcoholic Fatty Liver Disease in Rats by Stimulating miR-34a/SIRT1/FXR/p53 Axis. Sci Total Environ (2021) 784:147182. doi: 10.1016/j.scitotenv.2021.147182 34088068

[22] Lee DH Park SH Ahn J Hong SP Lee E Jang YJ. Mir214-3p and Hnf4a/Hnf4alpha Reciprocally Regulate Ulk1 Expression and Autophagy in Nonalcoholic Hepatic Steatosis. Autophagy (2021) 17(9):2415–31. doi: 10.1080/15548627.2020.1827779 PMC8496708 33078654

[23] Xu H Tian Y Tang D Zou S Liu G Song J. An Endoplasmic Reticulum Stress-MicroRNA-26a Feedback Circuit in NAFLD. Hepatology (2021) 73(4):1327–45. doi: 10.1002/hep.31428 32567701

[24] Tan L Xie Y Yuan Y Hu K. LncRNA GAS5 as miR-26a-5p Sponge Regulates the PTEN/PI3K/Akt Axis and Affects Extracellular Matrix Synthesis in Degenerative Nucleus Pulposus Cells. Vitro Front Neurol (2021) 12:653341. doi: 10.3389/fneur.2021.653341 34413821 PMC8369364

[25] Zhu C Zhang H Wei D Sun Z. Silencing lncRNA GAS5 Alleviates Apoptosis and Fibrosis in Diabetic Cardiomyopathy by Targeting miR-26a/B-5p. Acta Diabetol (2021) 58(11):1491–501. doi: 10.1007/s00592-021-01745-3 34091757

[26] Wang J Zhu Y Ni S Liu S. LncRNA GAS5 Suppressed Proliferation and Promoted Apoptosis in Laryngeal Squamous Cell Carcinoma by Targeting MiR-26a-5p and Modifying Ulk2. Cancer Manag Res (2021) 13:871–87. doi: 10.2147/CMAR.S250778 PMC7856352 33551645

[27] Avila DV Barker DF Zhang J McClain CJ Barve S Gobejishvili L. Dysregulation of Hepatic cAMP Levels *via* Altered Pde4b Expression Plays a Critical Role in Alcohol-Induced Steatosis. J Pathol (2016) 240(1):96–107. doi: 10.1002/path.4760 27287961 PMC4993672

[28] Guzman S Dragan M Kwon H de Oliveira V Rao S Bhatt V. Targeting Hepatic Kisspeptin Receptor Ameliorates Nonalcoholic Fatty Liver Disease in a Mouse Model. J Clin Invest (2022) 132(10):e145889. doi: 10.1172/JCI145889 35349482 PMC9106350

[29] Kota J Chivukula RR O'Donnell KA Wentzel EA Montgomery CL Hwang HW. Therapeutic microRNA Delivery Suppresses Tumorigenesis in a Murine Liver Cancer Model. Cell (2009) 137(6):1005–17. doi: 10.1016/j.cell.2009.04.021 PMC2722880 19524505

[30] Yan F Wang Q Lu M Chen W Song Y Jing F. Thyrotropin Increases Hepatic Triglyceride Content Through Upregulation of SREBP-1c Activity. J Hepatol (2014) 61(6):1358–64. doi: 10.1016/j.jhep.2014.06.037 25016220

[31] Wu L Mo W Feng J Li J Yu Q Li S. Astaxanthin Attenuates Hepatic Damage and Mitochondrial Dysfunction in non-Alcoholic Fatty Liver Disease by Up-Regulating the FGF21/PGC-1alpha Pathway. Br J Pharmacol (2020) 177(16):3760–77. doi: 10.1111/bph.15099 PMC7393201 32446270

[32] De Vincentis A Rahmani Z Muley M Vespasiani-Gentilucci U Ruggiero S Zamani P. Long Noncoding RNAs in Nonalcoholic Fatty Liver Disease and Liver Fibrosis: State-of-the-Art and Perspectives in Diagnosis and Treatment. Drug Discovery Today (2020) 25(7):1277–86. doi: 10.1016/j.drudis.2020.05.009 32439605

[33] Tao R Hu S Wang S Zhou X Zhang Q Wang C. Association Between Indel Polymorphism in the Promoter Region of lncRNA GAS5 and the Risk of Hepatocellular Carcinoma. Carcinogenesis (2015) 36(10):1136–43. doi: 10.1093/carcin/bgv099 26163879

[34] DiStefano JK Gerhard GS. Long Noncoding RNAs and Human Liver Disease. Annu Rev Pathol (2022) 17:1–21. doi: 10.1146/annurev-pathol-042320-115255 34416820 PMC9183788

[35] Lu S Su Z Fu W Cui Z Jiang X Tai S. Altered Expression of Long non-Coding RNA GAS5 in Digestive Tumors. Biosci Rep (2019) 39(1):BSR20180789. doi: 10.1042/BSR20180789 30606744 PMC6340949

[36] Ruan X Li P Ma Y Jiang CF Chen Y Shi Y. Identification of Human Long Noncoding RNAs Associated With Nonalcoholic Fatty Liver Disease and Metabolic Homeostasis. J Clin Invest (2021) 131(1):e136336. doi: 10.1172/JCI136336 PMC7773374 33048844

[37] Diraison F Moulin P Beylot M. Contribution of Hepatic *De Novo* Lipogenesis and Reesterification of Plasma non Esterified Fatty Acids to Plasma Triglyceride Synthesis During Non-Alcoholic Fatty Liver Disease. Diabetes Metab (2003) 29(5):478–85. doi: 10.1016/s1262-3636(07)70061-7 14631324

[38] Cohen CC Li KW Alazraki AL Beysen C Carrier CA Cleeton RL. Dietary Sugar Restriction Reduces Hepatic *De Novo* Lipogenesis in Adolescent Boys With Fatty Liver Disease. J Clin Invest (2021) 131(24):e150996. doi: 10.1172/JCI150996 34907907 PMC8670836

[39] Moore JB Gunn PJ Fielding BA. The Role of Dietary Sugars and *De Novo* Lipogenesis in non-Alcoholic Fatty Liver Disease. Nutrients (2014) 6(12):5679–703. doi: 10.3390/nu6125679 PMC4276992 25514388

[40] Wang Q Li S Jiang L Zhou Y Li Z Shao M. Deficiency in Hepatic ATP-Citrate Lyase Affects VLDL-Triglyceride Mobilization and Liver Fatty Acid Composition in Mice. J Lipid Res (2010) 51(9):2516–26. doi: 10.1194/jlr.M003335 PMC2918436 20488800

[41] Mao J DeMayo FJ Li H Abu-Elheiga L Gu Z Shaikenov TE. Liver-Specific Deletion of Acetyl-CoA Carboxylase 1 Reduces Hepatic Triglyceride Accumulation Without Affecting Glucose Homeostasis. Proc Natl Acad Sci U.S.A. (2006) 103(22):8552–7. doi: 10.1073/pnas.0603115103 PMC1570106 16717184

[42] Chirala SS Chang H Matzuk M Abu-Elheiga L Mao J Mahon K. Fatty Acid Synthesis is Essential in Embryonic Development: Fatty Acid Synthase Null Mutants and Most of the Heterozygotes Die. Utero Proc Natl Acad Sci U.S.A. (2003) 100(11):6358–63. doi: 10.1073/pnas.0931394100 PMC164451 12738878

[43] Zhu M Chen H Zhou S Zheng L Li X Chu R. Iron Oxide Nanoparticles Aggravate Hepatic Steatosis and Liver Injury in Nonalcoholic Fatty Liver Disease Through BMP-SMAD-Mediated Hepatic Iron Overload. Nanotoxicology (2021) 15(6):761–78. doi: 10.1080/17435390.2021.1919329 33961538

[44] Berger JM Moon YA. Increased Hepatic Lipogenesis Elevates Liver Cholesterol Content. Mol Cells (2021) 44(2):116–25. doi: 10.14348/molcells.2021.2147 PMC7941001 33658436

[45] Kartsoli S Kostara CE Tsimihodimos V Bairaktari ET Christodoulou DK. Lipidomics in non-Alcoholic Fatty Liver Disease. World J Hepatol (2020) 12(8):436–50. doi: 10.4254/wjh.v12.i8.436 PMC7475773 32952872

[46] Clugston RD Gao MA Blaner WS. The Hepatic Lipidome: A Gateway to Understanding the Pathogenes is of Alcohol-Induced Fatty Liver. Curr Mol Pharmacol (2017) 10(3):195–206. doi: 10.2174/1874467208666150817111419 26278391 PMC4757511

[47] Cesana M Cacchiarelli D Legnini I Santini T Sthandier O Chinappi M. A Long Noncoding RNA Controls Muscle Differentiation by Functioning as a Competing Endogenous RNA. Cell (2011) 147(2):358–69. doi: 10.1016/j.cell.2011.09.028 PMC3234495 22000014

[48] Ali O Darwish HA Eldeib KM Abdel Azim SA. miR-26a Potentially Contributes to the Regulation of Fatty Acid and Sterol Metabolism *In Vitro* Human HepG2 Cell Model of Nonalcoholic Fatty Liver Disease. Oxid Med Cell Longev (2018) 2018:8515343. doi: 10.1155/2018/8515343 30402207 PMC6196797

[49] He Q Li F Li J Li R Zhan G Li G. MicroRNA-26a-Interleukin (IL)-6-IL-17 Axis Regulates the Development of non-Alcoholic Fatty Liver Disease in a Murine Model. Clin Exp Immunol (2017) 187(1):174–84. doi: 10.1111/cei.12838 PMC5167023 27377869

[50] Ding D Ye G Lin Y Lu Y Zhang H Zhang X. MicroRNA-26a-CD36 Signaling Pathway: Pivotal Role in Lipid Accumulation in Hepatocytes Induced by PM2.5 Liposoluble Extracts. Environ pollut (2019) 248:269–78. doi: 10.1016/j.envpol.2019.01.112 30798028

[51] Di Sessa A Riccio S Pirozzi E Verde M Passaro AP Umano GR. Advances in Paediatric Nonalcoholic Fatty Liver Disease: Role of Lipidomics. World J Gastroenterol (2021) 27(25):3815–24. doi: 10.3748/wjg.v27.i25.3815 PMC8291022 34321846

[52] Luukkonen PK Qadri S Ahlholm N Porthan K Mannisto V Sammalkorpi H. Distinct Contributions of Metabolic Dysfunction and Genetic Risk Factors in the Pathogenesis of Non-Alcoholic Fatty Liver Disease. J Hepatol (2021) 76(3):526–535. doi: 10.1016/j.jhep.2021.10.013 34710482 PMC8852745

[53] Kleinboelting S van den Heuvel J Steegborn C. Structural Analysis of Human Soluble Adenylyl Cyclase and Crystal Structures of its Nucleotide Complexes-Implications for Cyclase Catalysis and Evolution. FEBS J (2014) 281(18):4151–64. doi: 10.1111/febs.12913 25040695

[54] Wang B Tsakiridis EE Zhang S Llanos A Desjardins EM Yabut JM. The Pesticide Chlorpyrifos Promotes Obesity by Inhibiting Diet-Induced Thermogenesis in Brown Adipose Tissue. Nat Commun (2021) 12(1):5163. doi: 10.1038/s41467-021-25384-y 34453052 PMC8397754

[55] Zhang WS Pan A Zhang X Ying A Ma G Liu BL. Inactivation of NF-Kappab2 (P52) Restrains Hepatic Glucagon Response *via* Preserving PDE4B Induction. Nat Commun (2019) 10(1):4303. doi: 10.1038/s41467-019-12351-x 31541100 PMC6754499

[56] Zhang K Kim H Fu Z Qiu Y Yang Z Wang J. Deficiency of the Mitochondrial NAD Kinase Causes Stress-Induced Hepatic Steatosis in Mice. Gastroenterology (2018) 154(1):224–37. doi: 10.1053/j.gastro.2017.09.010 PMC5742027 28923496

[57] Herzig S Hedrick S Morantte I Koo SH Galimi F Montminy M. CREB Controls Hepatic Lipid Metabolism Through Nuclear Hormone Receptor PPAR-Gamma. Nature (2003) 426(6963):190–3. doi: 10.1038/nature02110 14614508

[58] Tai CC Chen CY Lee HS Wang YC Li TK Mersamm HJ. Docosahexaenoic Acid Enhances Hepatic Serum Amyloid A Expression *via* Protein Kinase A-Dependent Mechanism. J Biol Chem (2009) 284(47):32239–47. doi: 10.1074/jbc.M109.024661 PMC2781636 19755416

[59] Sang L Ju HQ Yang Z Ge Q Zhang Z Liu F. Mitochondrial Long non-Coding RNA GAS5 Tunes TCA Metabolism in Response to Nutrient Stress. Nat Metab (2021) 3(1):90–106. doi: 10.1038/s42255-020-00325-z 33398195

[60] Piccinin E Villani G Moschetta A. Metabolic Aspects in NAFLD, NASH and Hepatocellular Carcinoma: The Role of PGC1 Coactivators. Nat Rev Gastroenterol Hepatol (2019) 16(3):160–74. doi: 10.1038/s41575-018-0089-3 30518830

[61] Lin J Handschin C Spiegelman BM. Metabolic Control Through the PGC-1 Family of Transcription Coactivators. Cell Metab (2005) 1(6):361–70. doi: 10.1016/j.cmet.2005.05.004 16054085

[62] Spiegelman BM Heinrich R. Biological Control Through Regulated Transcriptional Coactivators. Cell (2004) 119(2):157–67. doi: 10.1016/j.cell.2004.09.037 15479634

[63] Aharoni-Simon M Hann-Obercyger M Pen S Madar Z Tirosh O. Fatty Liver Is Associated With Impaired Activity of PPARgamma-Coactivator 1alpha (PGC1alpha) and Mitochondrial Biogenesis in Mice. Lab Invest (2011) 91(7):1018–28. doi: 10.1038/labinvest.2011.55 21464822

[64] Finck BN Gropler MC Chen Z Leone TC Croce MA Harris TE. Lipin 1 is an Inducible Amplifier of the Hepatic PGC-1alpha/PPARalpha Regulatory Pathway. Cell Metab (2006) 4(3):199–210. doi: 10.1016/j.cmet.2006.08.005 16950137

[65] Song S Attia RR Connaughton S Niesen MI Ness GC Elam MB. Peroxisome Proliferator Activated Receptor Alpha (PPARalpha) and PPAR Gamma Coactivator (PGC-1alpha) Induce Carnitine Palmitoyltransferase IA (CPT-1a) *via* independent gene elements. Mol Cell Endocrinol (2010) 325(1-2):54–63. doi: 10.1016/j.mce.2010.05.019 20638986 PMC3160239

