# Supplementary material for: LncRNA GAS5 Knockdown Mitigates Hepatic Lipid Accumulation via Regulating MiR-26a-5p/PDE4B to Activate cAMP/CREB Pathway
Source: Front Endocrinol (Lausanne). 2022 Jul 26;13:889858. doi: 10.3389/fendo.2022.889858 (PMC9361042; doi:10.3389/fendo.2022.889858)
Supplement: Supplementary file 1 [file DataSheet_1.docx]

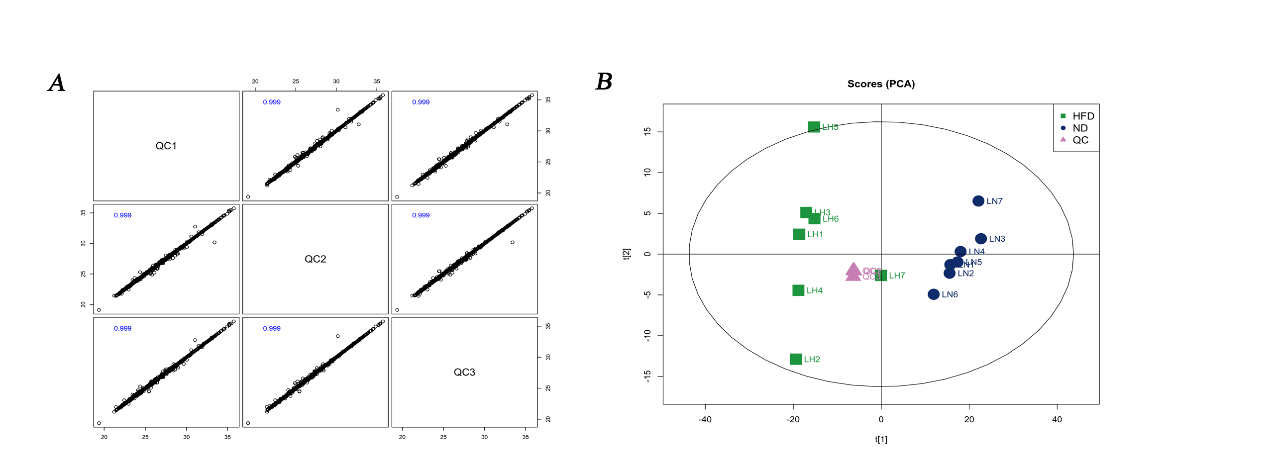


Figure S1. The stability and reproducibility of relative quantitative lipidome analysis. The correlation map of QC samples (A). Principal component analysis of HFD, ND and QC samples (B).

Pearson Correlation Analysis was performed to verify the stability and repeatability of the instruments, and the experimental results show that the correlation coefficients between QC samples are all above 0.9, indicating the high quality of experimental repeatability (Supplementary 1A). In addition, the close aggregation of QC samples also reflected the good repeatability of the experiment.


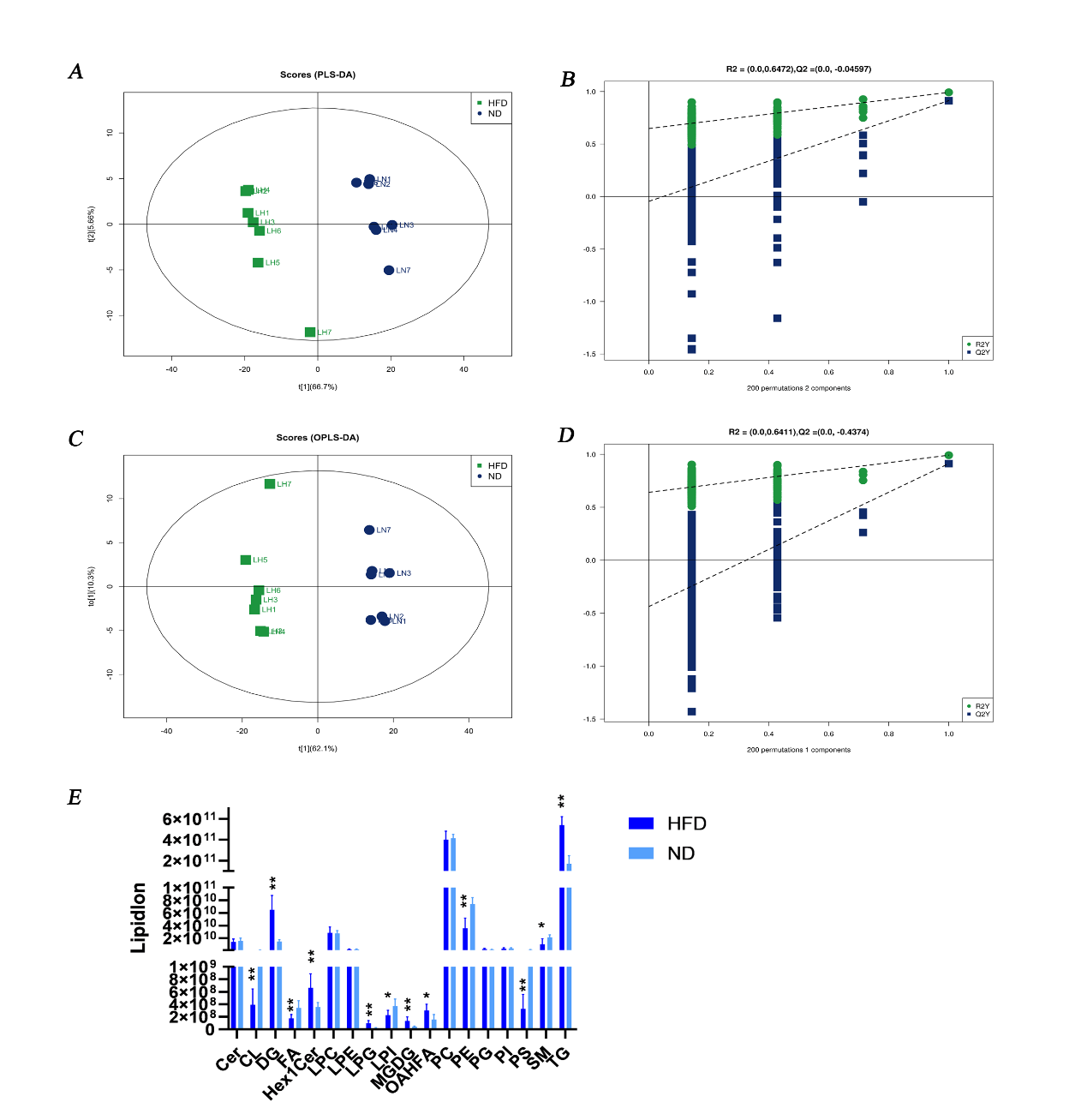


Figure S2. Significant changes of hepatic lipids between HFD and ND groups. Partial Least Squares Discrimination Analysis (PLS DA) (A), and Permutation test of PLS DA (B). Orthogonal Partial Least Squares Discrimination Analysis (OPLS DA) (C), and Permutation test of OPLS DA (D). 18 lipid classes with significantly different lipid molecules (OPLS DA VIP>1 and P value <0.05) detected with mass spectrometry (E).

For multivariate statistical analysis, PLS DA was used to establish a model of the relationship between lipid expression and sample category. The dots distribution reflected the degree of difference between and within groups. There was a significant difference between the tissue samples of mice fed by HFD and those fed by ND (A). Permutation testing were used to evaluate the robustness of the model. The value of R^2^ and Q^2^ in the random models gradually decreased with the downregulation of replacement retention, indicating there existed no over-fitting phenomenon (B). In OPLS DA, difference between HFD and ND samples was significantly verified by their t [1] values (C). In addition, the over-fitting phenomenon was excluded by the result of permutation test (D). The lipid concentrations of TG, DG, Hex1Cer, LPG, MGDG, OAHFA were significantly higher in HFD fed mice, while CL, FA, LPI, PE, PS, and SM were reduced in HFD fed mice. These results emphasized significant changes in hepatic lipids in the HFD groups.


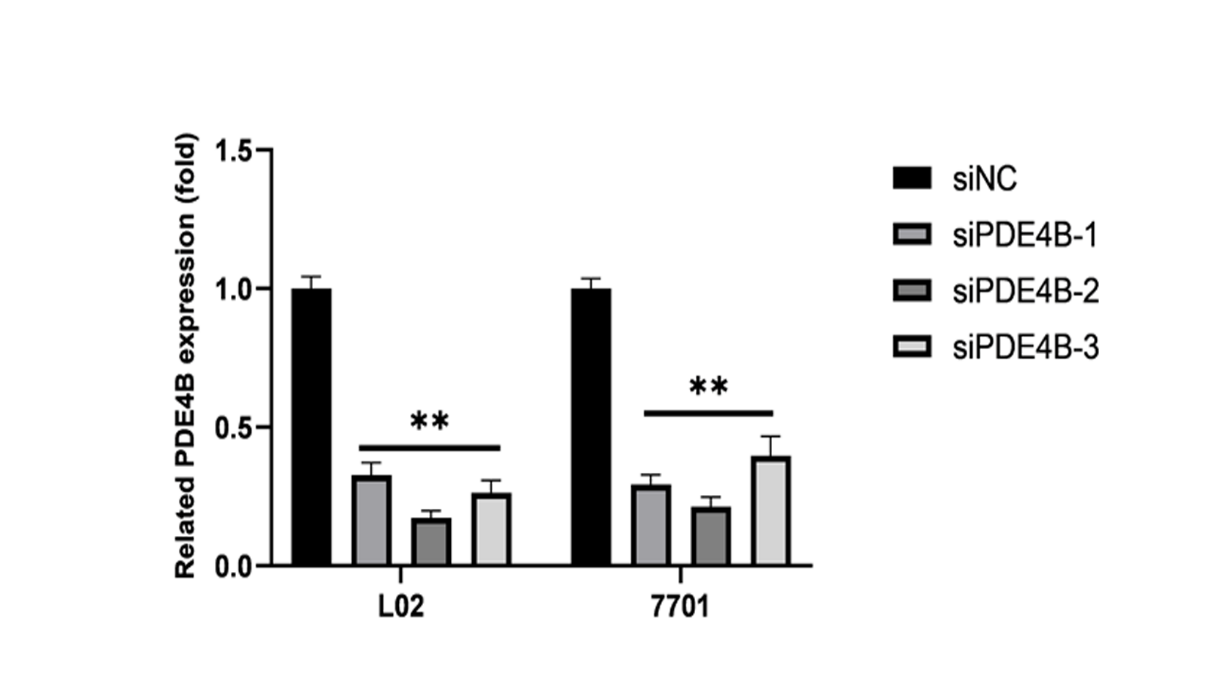
Figure S3. The expression of PDE4B in cells with three small interfering PDE4Bs transfection.

All three small interfering RNA for PDE4B (siPDE4Bs) significantly reduced the expression of PDE4B in L02 or 7701 cells. In both cell lines, siPDE4B-2 had the strongest inhibitory effects on cells.


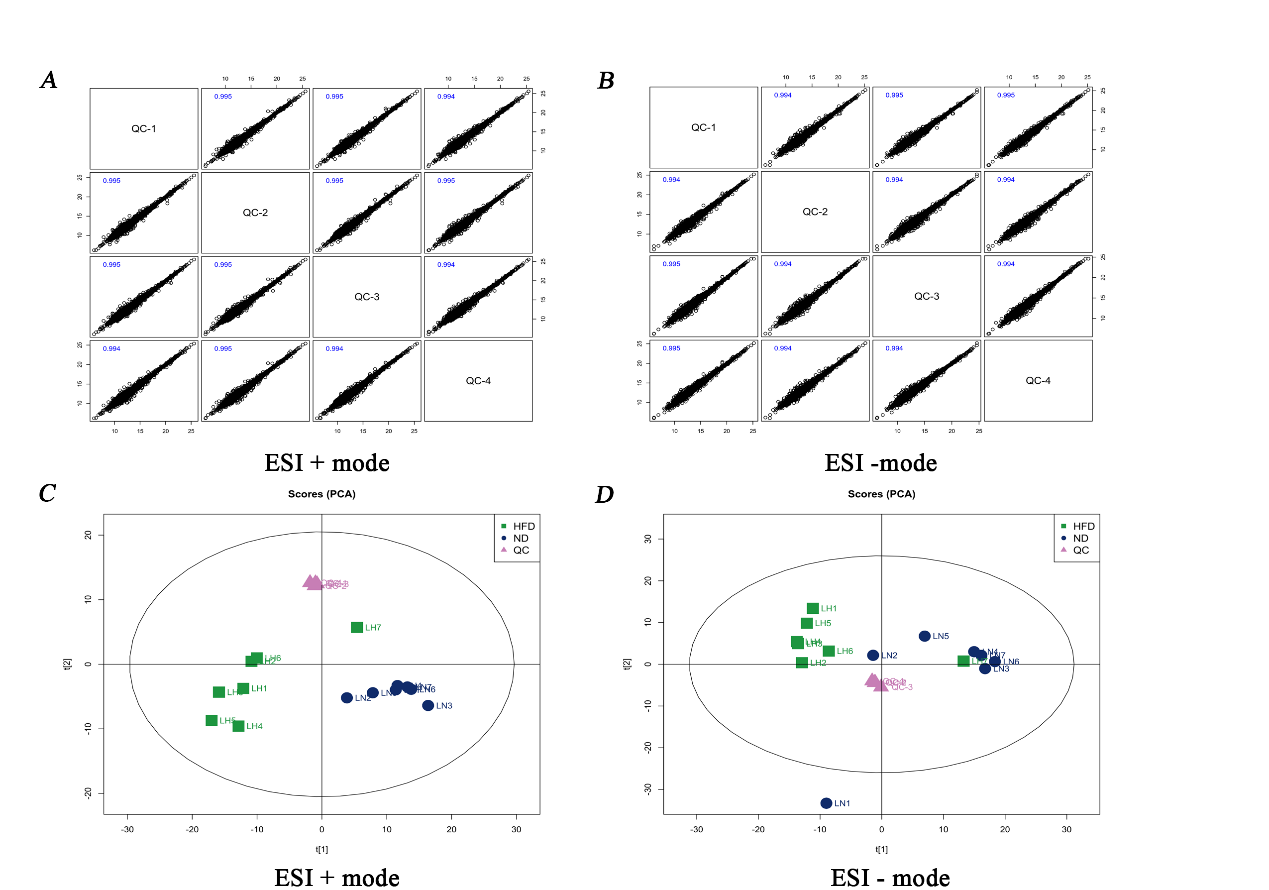


Figure S4. The stability and reproducibility of untargeted metabolomics analysis. The correlation map of QC samples in ESI + mode (A) and ESI – mode (B). Principal component analysis of HFD, ND and QC samples in ESI + mode (C) and ESI – mode (D).

The correlation coefficient greater than 0.9 in the ESI + mode (A) and ESI – mode (B), and the close aggregation of the QC samples (C and D) confirm the repeatability and stability of the experiment.


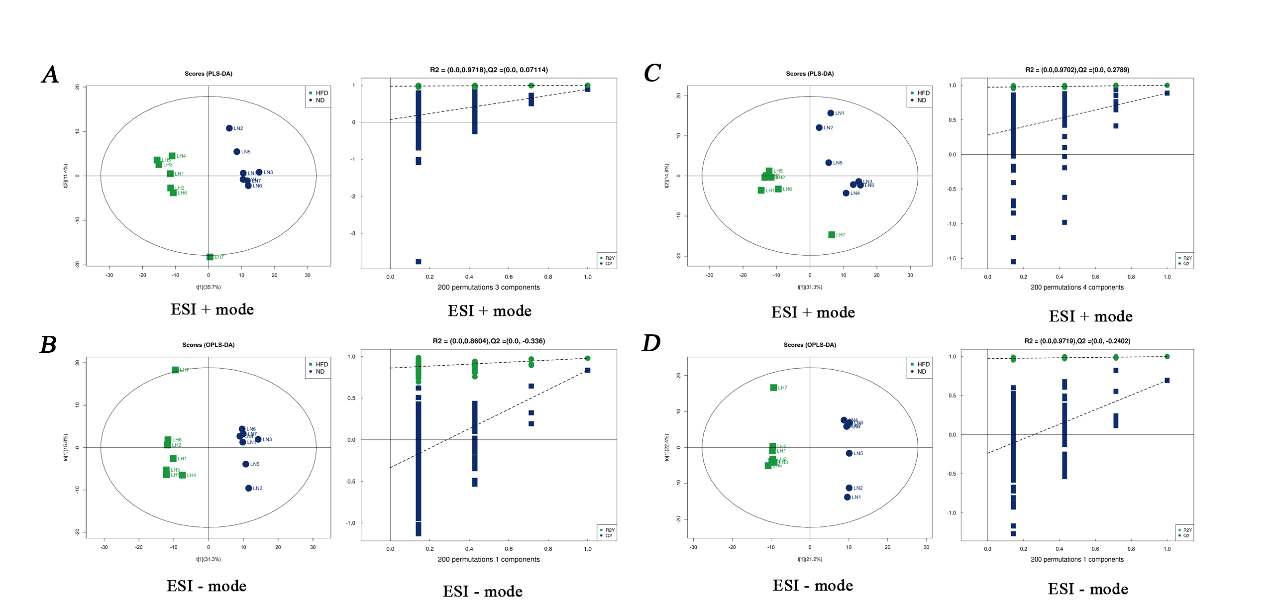


Figure S5. Significant changes of hepatic metabolites between HFD and ND groups. Partial Least Squares Discrimination Analysis (PLS DA), and Permutation test of PLS DA in ESI + mode (A), and ESI – mode (B). Orthogonal Partial Least Squares Discrimination Analysis (OPLS DA), and Permutation test of OPLS DA in ESI + mode (C), and ESI – mode (D).

Given the dispersion of the dots, the metabolites of the HFD groups are significantly different from those of the ND groups in both ESI + mode (A) and SEI – mode (B). There also existed a clear demarcation between the value of t [1] of the HFD group and the ND group in both ESI + mode (C) and SEI – mode (D), indicating a significant metabolites variation after HFD treatment. The robustness of the models was verified by the permutation testing.


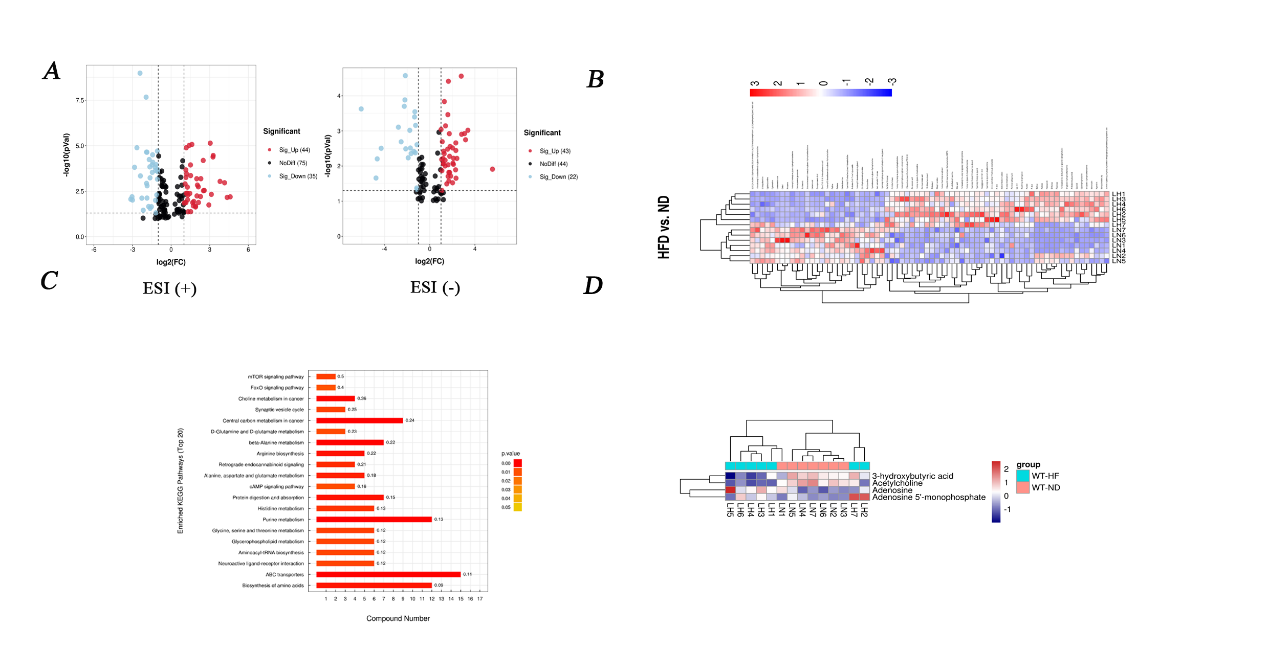


Figure S6. Untargeted metabolomics analysis of HFD mice vs. ND mice. Volcano plots of lipids and lipid-like metabolites with significant changes (fold change >2 or <0.5) of HFD and ND fed mice in electrospray ionization (ESI) positive and negative modes (A). Heatmap of significant different lipids and lipid-like metabolites (VIP>1 and p-value < 0.05) (B). Top 20 enriched KEGG pathways of hepatic metabolites when compared the high fed diet (HFD) with normal chow diet (ND) mice (C). Changes of hepatic metabolites involved in the cAMP pathway between the HFD and ND groups (D).

The concentration of lipids and lipid-like metabolites changed both in electrospray ionization (ESI) positive and negative modes (Figure 6A), and the pattern of metabolites variation was significantly different between HFD and ND fed mice (Figure 6B). 20 top KEGG pathways were shown and included cAMP pathway (Figure 6C). Metabolites involved in the cAMP pathway were different between HFD and ND groups (Figure 6C).
